# Biocementation mediated by native microbes from Brahmaputra riverbank for mitigation of soil erodibility

**DOI:** 10.1038/s41598-021-94614-6

**Published:** 2021-07-27

**Authors:** Anant Aishwarya Dubey, K. Ravi, Abhijit Mukherjee, Lingaraj Sahoo, Moses Akindele Abiala, Navdeep K. Dhami

**Affiliations:** 1 Indian Institute of Technology, Guwahati, 781039 India; 2 Curtin University, Perth, WA 6152 Australia; 3 Mountain Top University, Prayer City, Nigeria

**Keywords:** Riparian ecology, Civil engineering, Bacteria

## Abstract

Riverbank erosion is a global problem with significant socio-economic impacts. Microbially induced calcite precipitation (MICP) has recently emerged as a promising technology for improving the mechanical properties of soils. The present study investigates the potential of selectively enriched native calcifying bacterial community and its supplementation into the riverbank soil of the Brahmaputra river for reducing the erodibility of the soil. The ureolytic and calcium carbonate cementation abilities of the enriched cultures were investigated with reference to the standard calcifying culture of *Sporosarcina pasteurii* (ATCC 11859). 16S rRNA analysis revealed Firmicutes to be the most predominant calcifying class with *Sporosarcina pasteurii* and *Pseudogracilibacillus auburnensis* as the prevalent strains*.* The morphological and mineralogical characterization of carbonate crystals confirmed the calcite precipitation potential of these communities. The erodibility of soil treated with native calcifying communities was examined via needle penetration and lab-scale hydraulic flume test. We found a substantial reduction in soil erosion in the biocemented sample with a calcite content of 7.3% and needle penetration index of 16 N/mm. We report the cementation potential of biostimulated ureolytic cultures for minimum intervention to riparian biodiversity for an environmentally conscious alternative to current erosion mitigation practices.

## Introduction

The banks along the worlds’ mega-rivers are susceptible to land degradation due to severe soil erosion^1^. The natural causes of soil erosion include frequent changes in hydrological conditions, including strong river currents, intense rainfall, and climate change^2,3^. The land degradation imparts enormous socio-economical vulnerability to the residents of the riparian zone. Human interventions such as deforestation and non-engineered construction are further worsening the stability of the riverbanks^4^. In India, severe land degradation issues are confronted at the banks of Brahmaputra River, one of the top ten rivers by discharge in the world^5^.

The existing riverbank erosion control practices are inclined towards rigid structures such as aprons, gabions, and check dams, which can irretrievably damage the riparian ecology by producing an imbalance in sediment inflow and outflow^4^. These structures may drastically impact the features of the river channel and induce floods downstream^3,4^. Alternatively, riverbank erosion can be controlled with chemical grouting. However, synthetic grout materials, including micro-fine cement, epoxy, and silicates have been reported to be toxic for the geo-environment^6^ and, therefore, can negatively impact the flora, fauna, and crop productivity of the soil of the riparian zone. One of the options as a sustainable erosion mitigation technique is vegetation along the bank. However, vegetation and their effect on the erodibility of soil are difficult to comprehend and engineer due to their transient life cycle and complex root structures, which depend on the vegetation type, nutrition present in the soil, and the surrounding climate^7,8^. Therefore, an alternative riverbank erosion mitigation strategy with minimum intervention to the riparian ecology is urgently needed.

Nature has been forming sustainable cement for millions of years, as seen in the case of corals, beach rocks, anthills, and cave speleothems^9,10^. Within this context, microbial induced calcite precipitation (MICP) is proposed by several studies as a potential tool for ecologically sustainable ground improvement technology^11,12^. Previous studies have reported biogenic crustal formations in different soil environments, including marine (entisols and alfisols), desert (aridisols), forest, and organic soils (ultisols and histosols)^10,13^. To mimic the natural biocementation, most of the studies have employed urease-producing microbe in different kinds of soil; however, most of the studies are limited to fine sands due to the required pore throat size for free microbial movement^6^. The principle of MICP is to utilize the urease-producing bacteria to hydrolyze urea, as shown in Eq. (1). In the presence of any ionic calcium source, calcite is precipitated in the soil pore network to bind the cohesion-less granular soil particles^6,14^ as summarized in Eq. (2).

1 $${\text{CO}}\left( {{\text{NH}}_{2} } \right)_{2} + {\text{H}}_{2} {\text{O}}\mathop{\longrightarrow}\limits^{Microbial Urease}2{\text{NH}}_{4}^{ + } + {\text{CO}}_{3}^{2 - }$$

2 $${\text{Ca}}^{2 + } + {\text{CO}}_{3}^{2 - } \to {\text{CaCO}}_{3} \downarrow$$

The precipitated calcium carbonate in the soil pores bridges the sand grains to reduce the soil erodibility. There are certain advantages of MICP over existing grouting practices, such as easy permeation through soil media due to the water-like viscosity of the cementation solution and comparatively negotiable influence on the geo-environment^6,9^. The limitations associated with its prospective field application are ammonium production as a byproduct, non-uniformity of precipitation, and transport of healthy ureolytic bacteria in large quantities to the site^6,12^. While the potential strategies to negate a higher concentration of ammonia and non-uniformity are being investigated comprehensively^15–20^, the transport of calcifying bacterial culture to the desired site is largely unaddressed. The presence of native ureolytic bacteria and their enrichment on-site can tackle the challenge of bacteria transport^21,22^.

There are limited studies on soil erosion mitigation with MICP; however, most of these studies are based on the bio-augmentation approach with foreign bacteria *Sporosarcina pasteurii* (ATCC 11859). Salifu et al*.*^23^ reported the effect of tidal cycles on different soil slope angles treated with 18 cycles of MICP treatment solution of 0.7 M cementation solution. The study reported that calcite filled in 9.9% pore volume was effective in controlling soil erosion against 30 tidal cycles on the steep slope (53°). Jiang and Soga^24^ investigated seepage-induced internal erosion for gravel-sand mix in a novel designed device considering *Sporosarcina pasteurii* (ATCC 6452) and reported cementation concentration higher than 0·4 M reduce the erosion to a negligible level. Wang et al*.*^25^ investigated soil erosion in hydraulic flume and erosion function apparatus (EFA) considering PVA-based modified cementation solution and observed non-uniform calcite precipitation with 1 M cementation solution via surface percolation strategy. The study suggested spraying of biocementation solutions as an alternative viable application strategy. Later, Jiang et al*.*^26^ investigated rainfall-induced erosion simulations for soil treated with 0.2,1 and 2.0 M of cementation solution sprayed four times and observed that 0.2 and 1.0 M cementation solution treated soils were substantially more resistant against rainfall-induced erosion when compared to 2 M treated soil. One of the recent studies by Clarà Saracho et al*.*^27^ investigated the influence of tangential erosion by utilizing erosion function apparatus (EFA) and reported that treatment of soil sample with one pore volume of 0.08 M biocementation solution for 10 days brought erosion values down to a negligible level for a tangential flow of 0 to 0.185 m/s^27^. To the authors’ best knowledge, no study for riverbank erosion mitigation with a bio-stimulation approach has been reported yet.

As soil is rich in microbial diversity with approximately 10^9^–10^12^ microorganisms per kilogram of soil nearby the ground surface^28^, any supplemented foreign bacteria has to compete with the native microorganisms for their survival in the new environment^21,22,29^. Hence, utilizing the indigenous microorganisms over the bio-augmentation approach for soil improvement has definite advantages in terms of minimum intervention to native biodiversity. However, with the conventional biostimulation approach, there is a likelihood of stimulating the undesired and non-participating bacterial communities, which may slow down the biocementation process^30^. Moreover, with conventional stimulation, it is necessary to identify and profile the enriched communities, as there is a threat of stimulating pathogenic ureolytic bacteria such as *Mycobacterium tuberculosis,* which are reported to be present in natural soils^31^*.* Therefore, an approach with selective stimulation of ureolytic microorganisms from the indigenous biodiversity is highly necessitated*.*

With this background, we investigated the potential of the native bacterial communities of the Brahmaputra riverbank to control the erodibility of the riverbank soil by conducting (a) A selective enrichment of native ureolytic calcium carbonate precipitating microbial communities, (b) Isolation, identification, and characterization of the stimulated bacteria, (c). Comparison of their biocementation potential in terms of urease activity and calcium utilization rate with *Sporosarcina pasteurii* (SP), (d). Evaluation of strength improvement by MICP via selectively stimulating the isolated indigenous culture with the non-destructive needle penetration test, and (e). Investigation on the influence of incremental cementation level on the soil erodibility by simulating a bed slope in a newly configured laboratory-scale hydraulic flume test.

## Experimental summary

**Figure 1:**
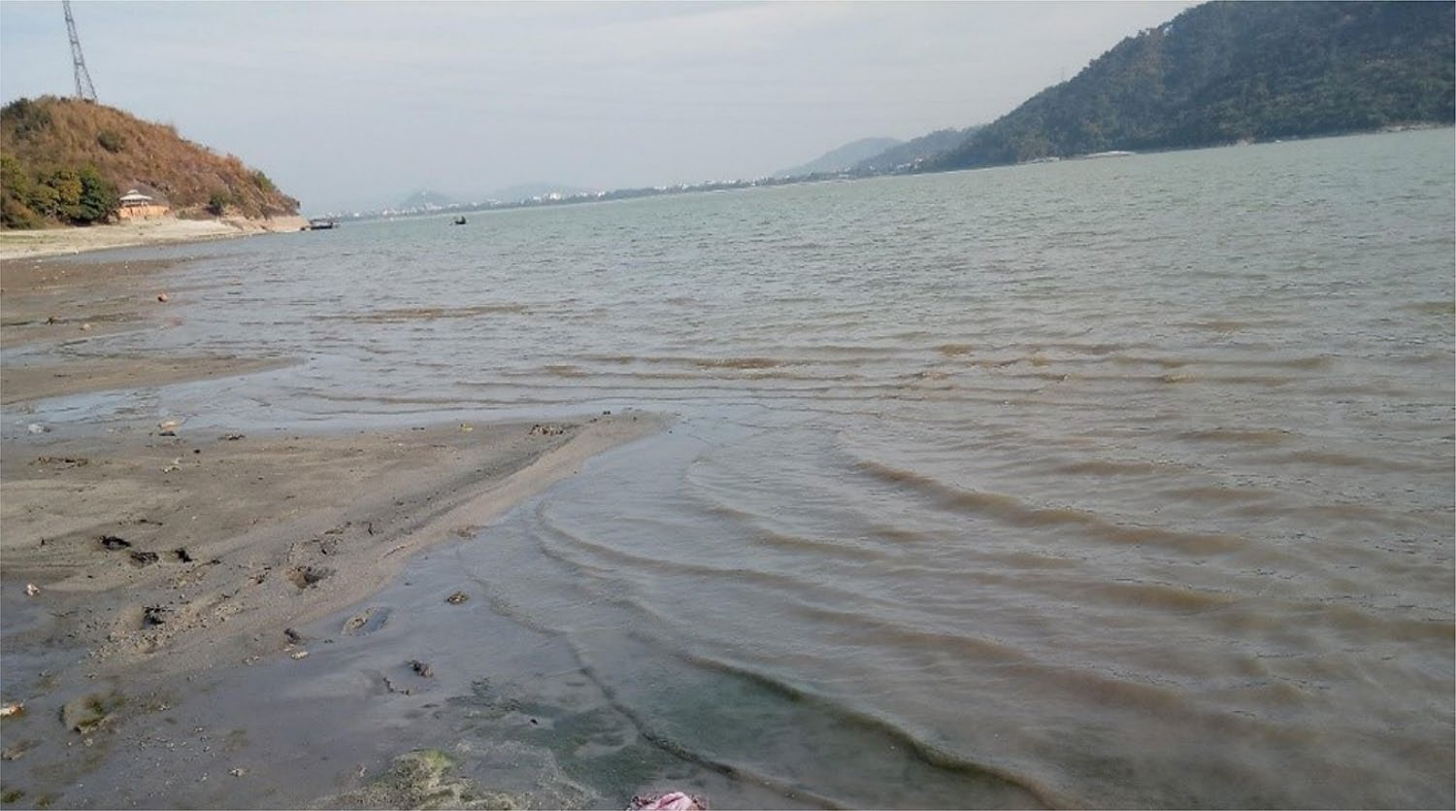
Soil collection site at Brahmaputra riverbank.

Soil samples were collected from the Brahmaputra riverbank (26° 10′ 50″ N; 91° 41′ 26″ E) near the Indian Institute of Technology, Guwahati, Assam, as shown in Fig. 1. The Brahmaputra river is an anabranching transboundary mega-river flowing in the south–Asian countries, including China, India, and Bangladesh^24^. Severe erosion with a rate of 80 square kilometers per year is reported at the Brahmaputra riverbank in the Assam valley along with the demolition of 2500 villages and 18 towns, impacting the livelihood of half a million people^32^. During the twentieth century (1912–1996), a total of 2358 square kilometer area from the Brahmaputra riverbank was eroded, and 1490 square kilometer of the area was accreted as fill, resulting in approximate 868 square kilometers of net land degradation in the Assam valley along the 630 km length of the Brahmaputra river^33^. Another study reported a 35.5% loss of land in the world’s largest river island, “Majuli” situated in the upper reach of the Brahmaputra river in Assam^5^. From 1997 to 2007, the rate of land loss accelerated, and 1057.5 square km was reported along the 750 km of the erosion-prone bank line^34^. Therefore, the protection of these banks is paramount for social, economic, and environmental sustainability. For comparison, another soil sample was collected from a nearby natural vegetative slope (26° 11′ 05″ N; 91° 41′ 32″ E) inside the Indian Institute of Technology, Guwahati campus in the vicinity of the riverbank. The topsoil (1 cm) was removed and the soil beneath was collected in a sterile tube from both sites and kept in an ice bucket for isolation purposes. The soil was collected separately for geotechnical classification within 50 cm depth. The engineering properties and physicochemical characterization of soil evaluated following American Society for Testing and Materials standards (ASTM D7503-18, D4972-19, D6913/D6913M-17, D854-14)^35–38^ and Soil Survey Staff (2014), Unites States Department of Agriculture (USDA) classification^39^ are illustrated in Table 1. As per the USCS (Unified Soil Classification System) the soil collected at Brahmaputra bank is characterized as poorly graded fine sand (SP), while the soil collected from nearby slope is identified as clayey sand (SC).

**Table 1:** Soil properties.

Properties	Site 1 (Brahmaputra riverbank)	Site 2 (natural slope near IIT Guwahati-core 4)
Coordinates	26° 10′ 50″ N; 91° 41′ 26″ E	26° 11′ 05″ N; 91° 41′ 32″ E
pH	7.5	8
Electrical conductivity (µS/cm)	8	22
Cation exchange capacity (meq/100 g)	0	10
Predominant minerals	Quartz, mica	Quartz, hematite, kaolinite
Environmental temperature	32 ± 3 °C	32 ± 3 °C
Specific gravity	2.7	2.65
Coarse sand content% (2 mm–4.75 mm)	0	1
Medium sand content% (0.425–2 mm)	1	26
Fine sand content% (0.075–0.425 mm)	94	54
Silt content % (0.002–0.075 mm)	5	14
Clay content % (≤ 0.002 mm)	0	5
Coefficient of uniformity (C_u_)	1.67	18.75
Coefficient of curvature (C_c_)	1.06	4.68
USCS classification	SP	SC
USDA classification	Sand	Loamy sand
Taxonomy	Entisols	Ultisols
Maximum dry density (kN/m^3^)	15.36	16.75

**Figure 2:**
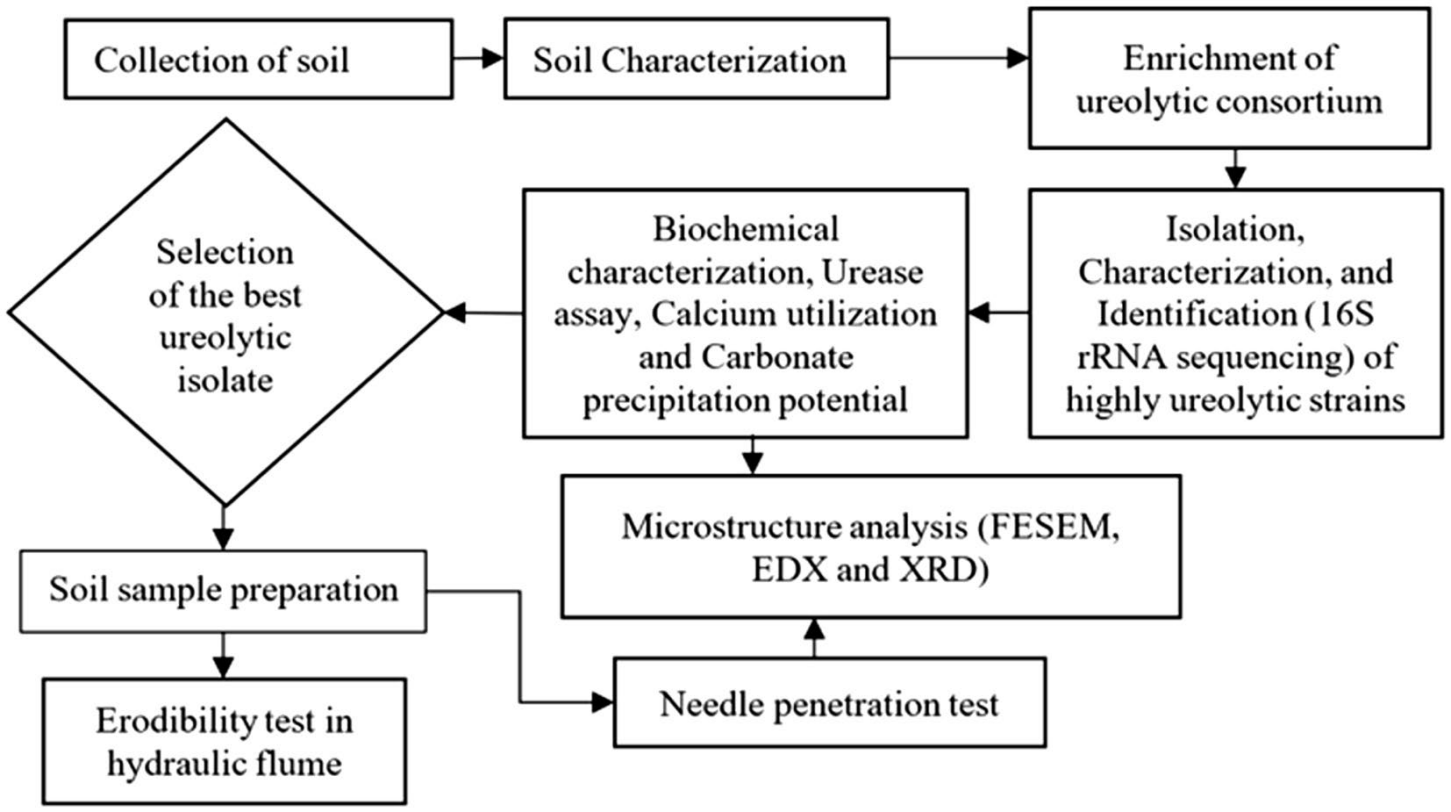
Schematic for the experiments in the current study.

After collecting the soil, a series of experiments were performed to enrich, isolate, and characterize the biocementation potent microorganisms. A summary of the work-plan can be comprehended by the flow chart of Fig. 2. After the isolation and characterization of the native bacteria, the best isolate was selected for soil treatment. The treated soil specimens with the best performing ureolytic strain were evaluated by the needle penetration resistance and hydraulic flume tests. The microstructural analysis of the precipitates and the treated soil sample was conducted to evaluate the morphology and mineralogy of the precipitates and the treated soil.

## Results and discussion

### Biostimulation of ureolytic communities

#### Enrichment

The native communities of the soils were successfully grown in the enrichment media (NB5U). The cultivated communities after two subcultures were serially diluted (10^–2^ to 10^–6^) and were spread with a sterile loop over Nutrient agar plates supplemented with 2% urea. Later, 36 morphologically distinct single colonies were obtained on the urea agar base plates based on visual observation.

#### Isolation, identification, and characterization of the ureolytic isolates

Out of 36 isolated bacteria, six isolates (BS1, BS2, BS3, BS4, LS1, and LS2) were selected after checking for the urease activity test on the urea agar base (UAB) plate. These selected isolates turned the color of the UAB plate from orange to pink within 12 h. The 16 s rRNA sequence revealed the isolates as relatives of *Sporosarcina pasteurii* (SP). The details of the identified isolates are provided by NCMR (details in Supplementary Table 1). The biochemical characterization (details in Supplementary Table 2) of the isolates revealed that all the isolates are Gram-positive. All the isolates were rod-shaped, endospore-forming, urease, and oxidase positive. All the isolates were not able to utilize the Lysine and ONPG, contrary to SP.

**Figure 3:**
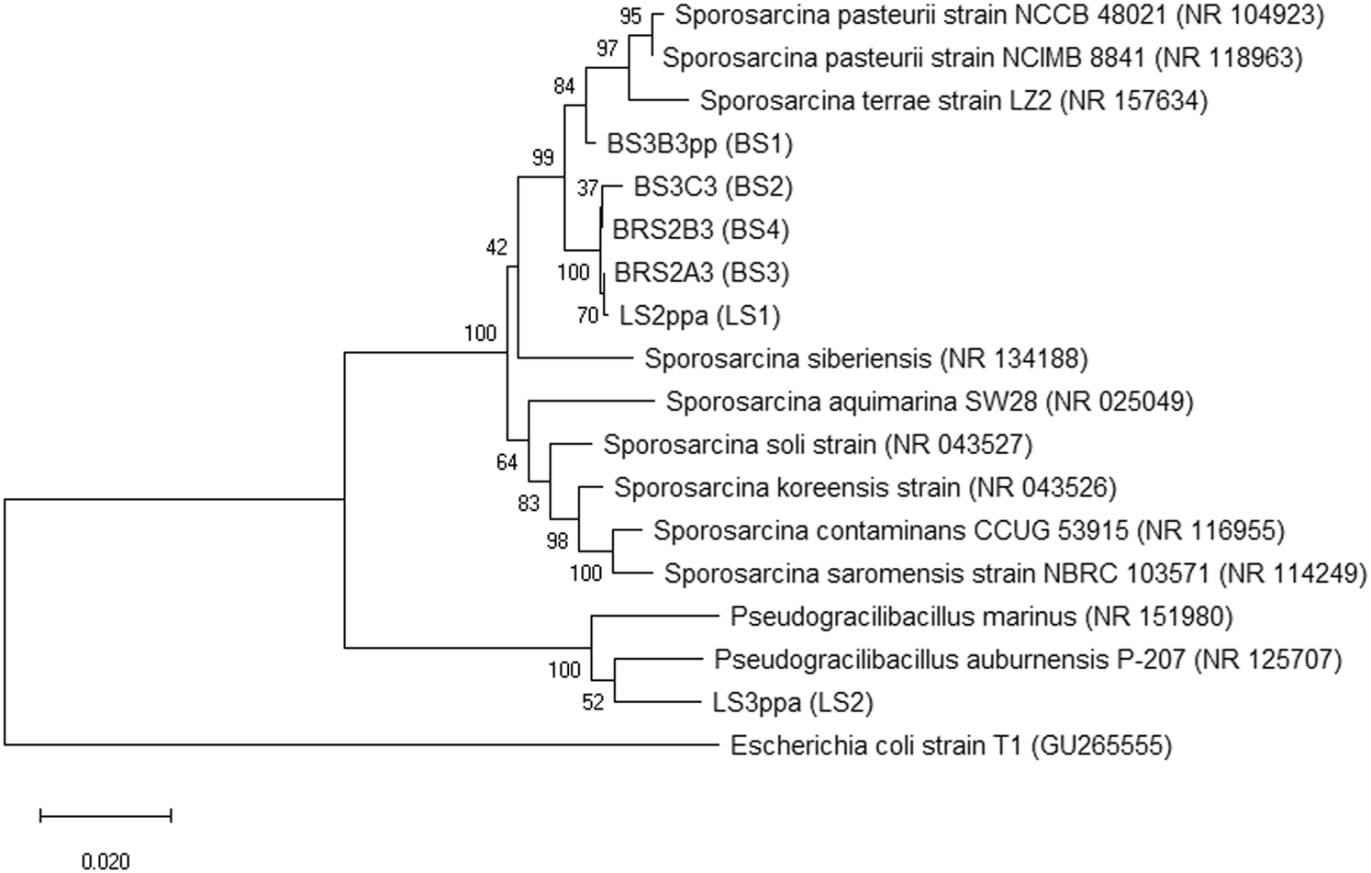
Neighbor-joining phylogenetic tree based on the 16S rRNA sequence of the isolates and reference sequence from the GenBank database (NCBI).

Further investigation of the isolated sequence was done via the NCBI database. The sequences were submitted to the GenBank database of the NCBI (National Center for Biotechnology Information) under the accession number MW024144 to MW024149. The BLAST analysis suggested that these strains are close relatives and indicate the possibility of being novel strains of the *Sporosarcina* family. We found that the isolate BS1 and BS2 had 96.62% (coverage 100%) and 96.22% (coverage 99%) identity with *Sporosarcina siberinisis* (NCBI accession number NR 134188). BS3 had 98.8% identity (coverage 97%) with *Sporosarcina pasteurii* (NCBI accession number NR 104923). BS4 and LS1 had 97.4% (coverage 99%) and 97.37% identity (coverage 100%) with *Sporosarcina soli* (NCBI accession number NR 043527). Contrarily, LS2 was found to be related to the *Pseudogracilibacillus* family. LS2 was observed to be closely related to *Pseudogracilibacillus auburnensis* P-207 with 97.06% identity (96% coverage). Based on these findings, the Phylogenetic tree was constructed with bootstrap (1000 replicates) considering the reference sequences obtained from the BLAST analysis, as shown in Fig. 3. The threshold criteria to differentiate two species is defined as 98.65% similarity score with the reference culture from databank^40^, while another study has suggested that in case of similarity index is > 99%, the unknown isolate should be assigned to a species, and if the unknown isolates have similarity score between 95 to 99% to a reference sequence, the isolate should be assigned to the genus^41^. However, further investigation is suggested to conclude if the reported strains are novel or merely mutants of the reference strains of the databank. Similar observations were made at Graddy et al*.*^22^*,* where the majority of the isolated strains (47 out of 57) from bio-stimulation soil tanks were found to be strains of the *Sporosarcina* genus*.* It is worth noting that the soil enrichment media for stimulation was rich in urea, similar to Gomez et al*.*^42^ and Graddy et al*.*^22^*,* which is conditional stress for selective stimulation of ureolytic microorganisms. Moreover, the isolated strains were screened based on morphology and qualitative urease activity.

### Evaluation of biocementation potential of the isolated strains

#### Growth and pH

**Figure 4:**
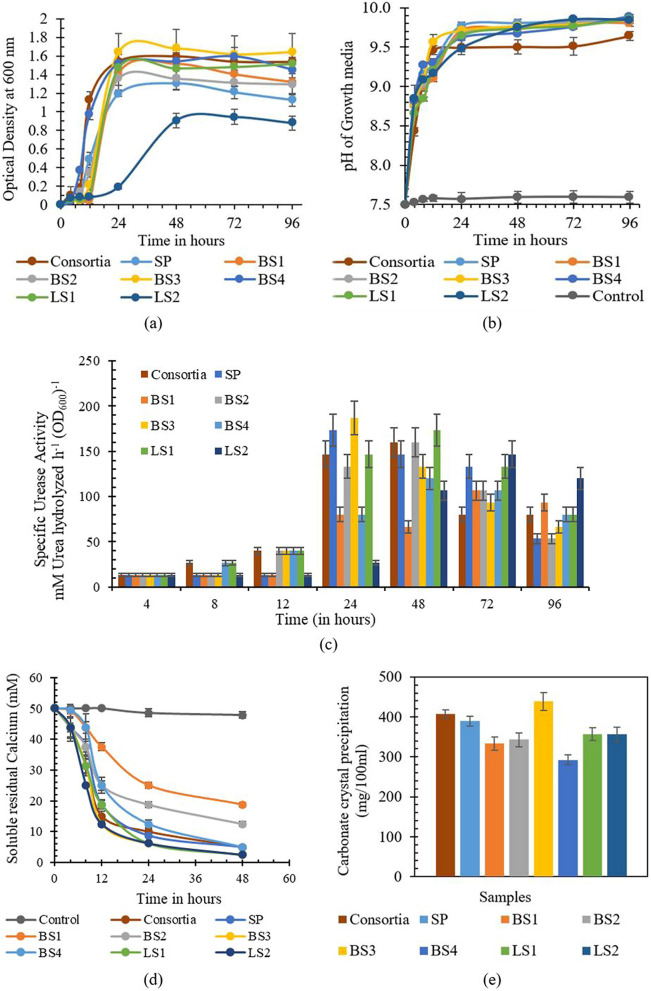
(**a**) Growth characteristics, (**b**) pH, (**c**) specific urease activity, (**d**) calcium utilization rate, and (**e**) carbonate precipitation rate of the isolates and consortia.

The various parameters of the biocementation potential of the isolated strains in comparison with *Sporosarcina pasteurii* (SP) have been plotted in Fig. 4. The growth characteristics of the isolates in NBU media and pH during growth have been represented in Fig. 4a and b. The initial pH of the growth media is kept at 7.5. It was observed that the pH of the growth media rises to 9.5 within 24 h of growth, indicating that these strains favor an alkaline environment to grow similar to SP^43^. All the isolates start growing when the pH of the media rises to 8.5 or above. Isolate LS2 was observed to have slower growth when compared with other isolates. This can be explained as LS2 belongs to different genera (*Pseudogracilibacillus)*.

#### Specific urease activity

The specific urease activities of the isolates were found to be comparable with SP (shown in Fig. 4c)***. ***Based on the provided NBU media and growth condition, the specific urease activity of SP is found to be 173.44 mM urea hydrolyzed h^−1^ (OD_600_)^−1^, which is around 2.9 mM urea hydrolyzed min^−1^ (OD_600_)^−1^. The specific urease activity of the isolate BS3 was observed to be maximum as 186.6 mM urea hydrolyzed h^−1^ (OD_600_)^−1^ during a growth period of 24 h and pH > 9. Consortia also demonstrated significant urease activity as 160 mM urea hydrolyzed h^−1^ (OD_600_)^−1^ at a growth period of 48 h. The maximum ureolytic activity in BS1 was observed after 72 h of growth with a value of 106.67 mM urea hydrolyzed h^−1^ (OD_600_)^−1^. Maximum specific urease activity of the isolate BS2, BS4, and LS1 was observed to be 160.2, 120, and 173.4 mM urea hydrolyzed h^−1^ (OD_600_)^−1^ respectively after a growth duration of 48 h. LS2 demonstrated the maximum specific urease activity of 146.4 mM urea hydrolyzed h^−1^ (OD_600_)^−1^. The observed order of specific urease activity at 24 h of growth period is BS3 > SP > Consortia > LS1 > BS2 > BS4 > LS2 > BS1. As the urease activity of the strains depends on the growth media, urea content, and environmental conditions such as pH and Temperature^44^, we considered the conditions at the riverbank at the time of isolation, and the pH and temperature of the growth media were set at 7.5 and 37 degrees Celsius. The specific urease measured by the electrical conductivity method is reported to be between 3 to 9.7 mM urea hydrolyzed min^−1^ (OD_600_)^−1^ in yeast-extract urea media at pH 7 and temperature 30 degrees Celsius^43^. It is reported around 5 mM urea hydrolyzed min^−1^ (OD_600_)^−1^ in the nutrient broth urea (2%) media at a temperature of 25 degrees Celsius^44^. The comparative analysis of the urease activity (measured by electrical conductivity method) was done considering SP as positive control in this study. The maximum specific urease activities of all isolates were found to be in a range of 106.67 to 186.67 mM urea hydrolyzed h^−1^ (OD_600_)^−1^ (1.78 to 3.11 mM urea hydrolyzed min^−1^ OD_600_^–1^), which indicates that all of the isolated strains are capable of biocementation^43,45^.

#### Calcium utilization and carbonate precipitation potential

It was experimentally observed that the depletion of the supplemented soluble calcium in the precipitation media (PM) was corresponding to the ureolytic activities of the isolated strains. Within 48 h of introducing 1% bacteria (OD600 = 1) in the precipitation media, the soluble calcium chloride (50 mM) was utilized to precipitate carbonate crystals, as illustrated in Fig. 4d. Within 12 h of the inoculation period, BS3 was able to utilize 75% of the supplied calcium, while SP was able to utilize only 62.5% of the soluble calcium. The order of the calcium utilization potential in the isolates was observed as BS3 ≥ LS2 > L.S.1 > Consortia > SP > BS4 > BS2 > BS1 during the inoculation period. Contrarily, LS2, despite being a comparatively slow urease-producing bacteria, was able to utilize calcium ions at par with other isolates. Negligible changes were observed in the soluble calcium concentration of the control group eliminating the possibility of abiotic precipitation.

The carbonate precipitation rate for each isolate (1% at OD_600_ = 1) for the 50 mM cementation media is plotted in Fig. 4e. The isolate BS3 with maximum ureolytic activity (specific urease activity 186.6 mM urea hydrolyzed min^−1^ OD_600_^–1^) precipitated the highest carbonate crystals after 96 h of the incubation period. BS3 precipitated 438 mg/100 ml of carbonate crystals, which is around 87.66% precipitation from the total supplied CaCl_2_, while precipitation with SP was quantified as 389 mg/100 ml (78%). The precipitation in consortia was observed to be 407 mg/100 ml (81%), which is slightly higher than SP. Precipitation in other isolates was found to be significantly lower than isolate BS3. Isolate BS1and BS2 precipitated 334 mg/100 ml (67%) and 343 mg/100 ml (69%) of carbonate crystals respectively, whereas isolate LS1 and LS2 precipitated around 357 mg/100 (71%) ml of carbonate crystals each. Isolate BS4 precipitated minimum carbonate crystals 292 mg/100 ml (58%). No precipitation was observed in the negative control set. Low concentrations of bacterial cells (1%) were considered in this experiment to slow down the urea hydrolysis in order to differentiate the calcium utilization potential of the isolated strains. This approach was modified from Dhami et al*.*^46^, and our results show agreement with their finding where 1% of SP cells depletes the 25 mM of CaCl_2_ within 24 h. It was observed that all the isolates took approximately 48 h to deplete the 50 mM CaCl_2_. The depletion of soluble calcium concentration was rapid in the initial 24 h in all the isolates. After 48 h, the residual soluble calcium was observed to be in the range of 2.5–5 mM in all the isolates (except BS1 and BS2), which might be due to loss of super-saturation caused by the unavailability of nutrient for bacterial cells to continue urea hydrolysis in the precipitation media^13,43,47^. The mineralogy of precipitated carbonate polymorphs (calcite, aragonite, vaterite) and the residual calcium are also influenced by pH, temperature, saturation index, dissolved organic carbon concentration, and the Ca^2+^ /CO_3_^2−^ratio along with the presence of metabolites in the precipitation media^13,47,48^. As maximum precipitation was recovered with the isolate BS3, the isolate BS3 was selected for further investigation on soil improvement.

#### Microstructure analysis of the precipitates

**Figure 5:**
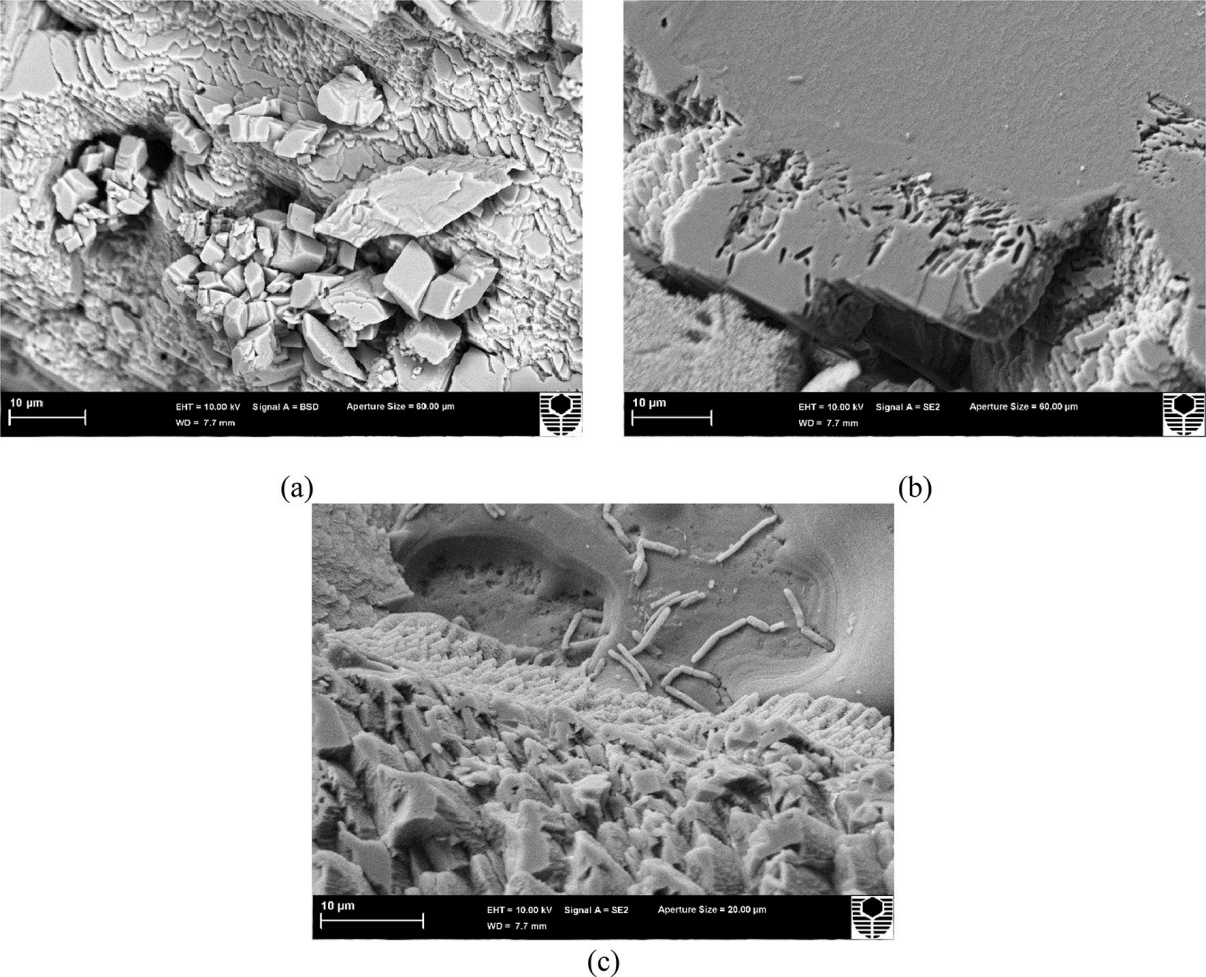
FESEM images of the calcite precipitated from BS3 (**a**) Coagulated crystals (**b**) Bacterial imprints, (**c**) Entrapped bacteria on the precipitates.

The FESEM images of the carbonate crystal precipitated from BS3 were investigated further. The shape of the precipitated crystals was observed to be rhombohedral and trigonal (Fig. 5a). The average size of the crystals was observed in a range of 25 to 50 microns. The entrapped bacteria and rod-shaped bacterial imprints were identified (Fig. 5b), indicating that the bacteria acted as a nucleation site^14^. The smaller crystals were observed to coagulate in layers to develop larger calcite crystals. The entrapped bacteria were noticed on the grown and coagulated calcite crystal in Fig. 5c. After taking the FESEM image (Fig. 5a) of the precipitate, EDX analysis was conducted, and the elemental composition suggested an abundance of calcium, carbon, and oxygen, which indicates the presence of calcium carbonate crystals (Supplementary Fig. 1). XRD analysis was conducted to confirm the mineralogy of the precipitates, and the majority of the observed peaks of the XRD plot belonged to calcite, which is consistent with the observation of rhombohedral crystal shapes in the FESEM image. The XRD analysis also suggested an insignificant presence of aragonite in the precipitates.

### Application of native communities on riverbank soil and its influence on soil strength

#### Needle penetration resistance of treated soil

**Figure 6:**
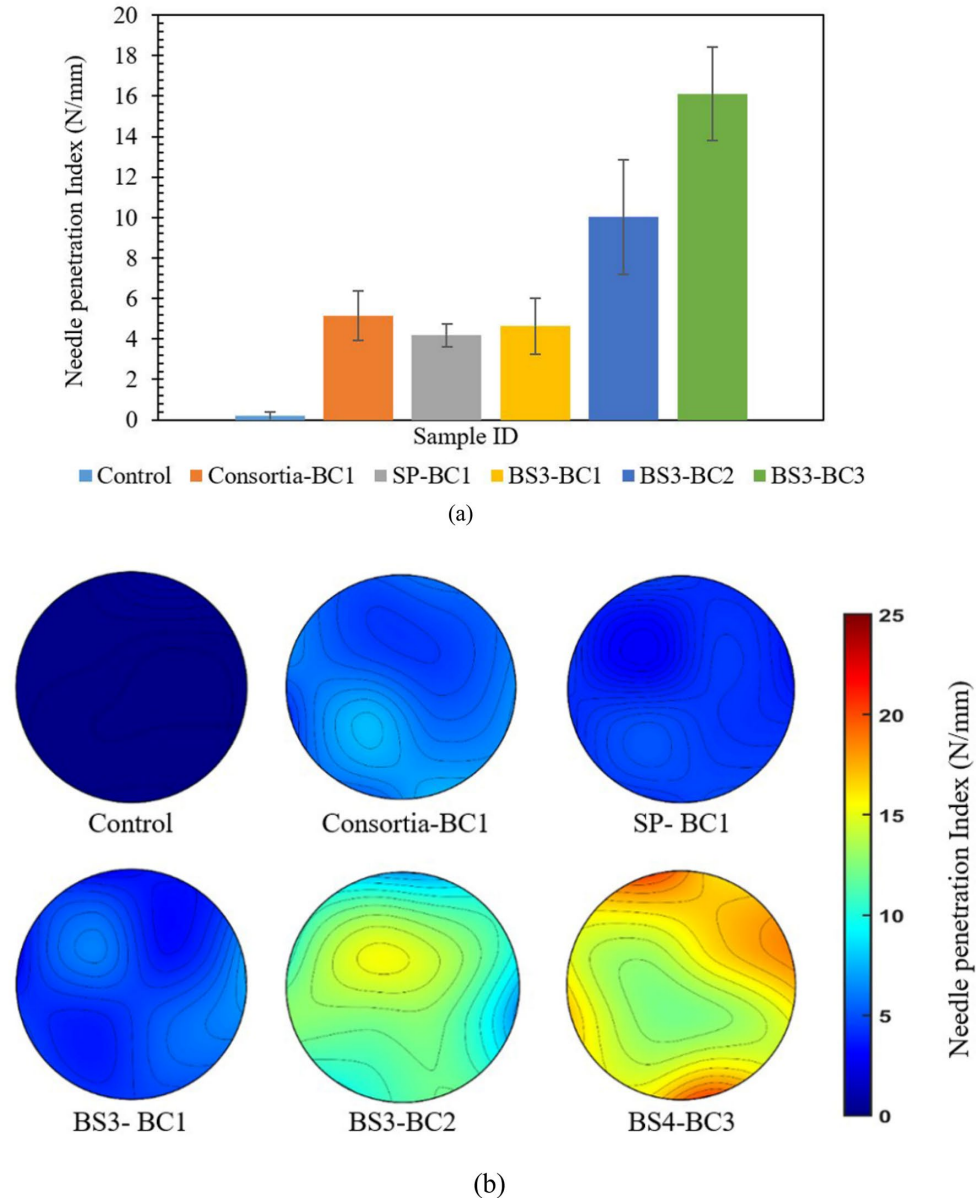
Comparison of the Needle penetration resistance (N/mm) of the treated soil specimen (**a**) average values and (**b**) the contours.

The average NPI (N/mm) for different cases has been shown in Fig. 6a. No notable resistance was observed in the loose untreated sand (control) against the needle penetration. With one bio cementation cycle treatment, the consortia-treated soil sample (Consortia-BC1) demonstrated a higher value of NPI (5.15 N/mm) than SP-BC1 (4.19 N/mm) and BS3-BC1 (4.64 N/mm). The increase in the biocementation cycle treatment significantly improved the needle penetration resistance. Sample BS3-BC2 showed 116% improvement with the NPI value of 10.03 N/mm when compared to one cycle treated sample BS3-BC1. A similar trend was observed in the sample BS3-BC3 (NPI = 16.12 N/mm), which showed around 347% improvement in NPI when compared to sample BS3-BC1. From the needle penetration test, it was evident that the penetration resistance of treated soil improves significantly with the increased level of biocementation cycles, indirectly indicating an improvement in the soil erodibility resilience. Since non-uniformity is one of the undesired traits of MICP, a contour was plotted corresponding to the 25 points NPI, as shown in Fig. 6b. The contrasting color difference in the contours of the samples BS3-BC1, BS3-BC2, and BS-BC3 clearly demonstrates the stark difference in the strength of treated samples. The non-uniformity in the strength of treated soil crust of sample BS3-BC2 and BS3-BC3 can also be realized with the contrasting color gradient of the NPI contours.

Since the rate of penetration has an insignificant influence on the test results, the needle penetration test is recommended by the International Society of Rock Mechanics (ISRM) for quick, non-destructive testing of the strength of the stabilized soils and soft rocks^49^. As a large number of tests can be conducted due to the small diameter of the needle without destroying the sample, the needle penetration test is a better alternative to evaluate the local grain bonding in the biocemented soil than bulk strength properties like unconfined compressive strength and calcite content. Another rationale for choosing needle penetration test over conventional soil strength evaluation tests was that a pocket type penetrometer could be developed with the configuration in the present study for non-destructive monitoring of the soil strength improvement with biocementation application in the field. The response of the needle penetration resistance in terms of nominal strain (ratio of penetration to rod diameter) also indicated that the measured responses are independent of needle diameter for a small range, i.e., 1 to 3 mm^49,50^. A portable penetrometer of Maruto. Co. ltd. (needle maximum diameter 0.84 mm at 12 mm from the tip) have been correlated with high confidence value to conventional physicochemical parameters such as unconfined compressive strength (UCS), elasticity modulus, and elastic wave velocity in several studies^50^. In our setup, we have utilized a similar configuration chenille 22 needle with (maximum diameter 0.86 mm at 9 mm from the tip) and a penetration rate of 15 mm/minute for measuring the strength properties of cemented soil. Adopting the UCS and NPI correlation suggested by Ulusay et al*.*^51^, the UCS of samples BS3-BC1, BS3-BC2, and BS3-BC3 are around 1.67 MPa, 3.4 Mpa, and 5.3 Mpa.

It is worth noting that in the needle penetration resistance tests, the boundary of the Petri dish can influence the test results. Therefore, trials were conducted and based on the findings, all penetrations were conducted at points at least five times the diameter of the needle away from the boundary to negate the influence of boundary conditions. The maximum penetration was conducted only up to 50% of the depth of prepared biocemented soil samples in the Petri dishes to avoid inference from the bottom of the Petri dish.

#### Erodibility test in the hydraulic flume

**Figure 7:**
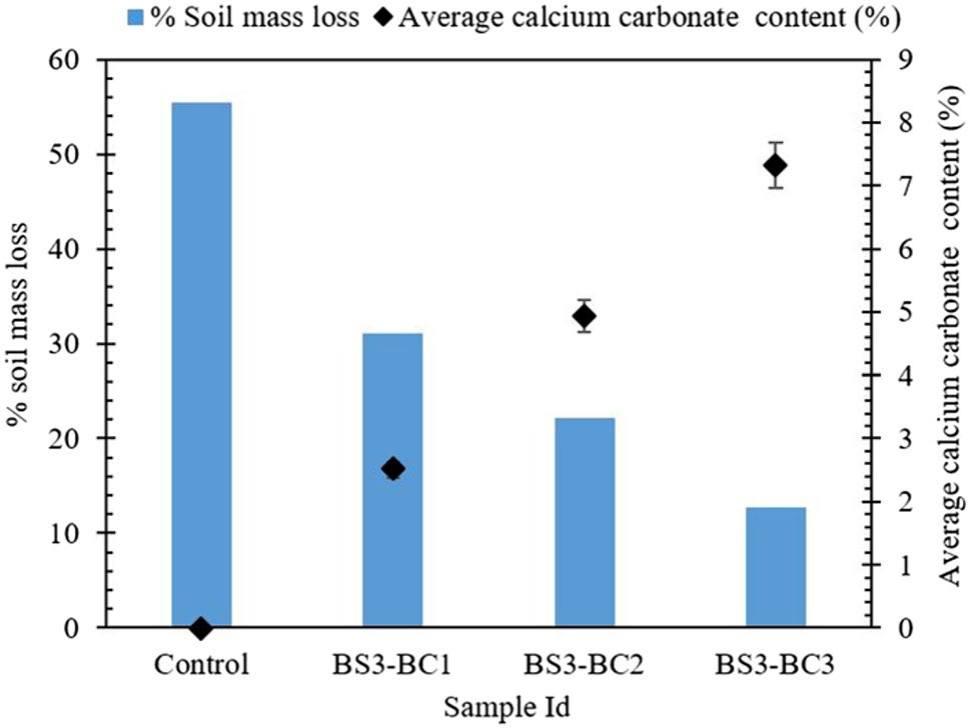
Weight of the eroded soil (%) after the hydraulic flume test.

To investigate the influence of hydraulic current on different levels of biocementation, all the treated samples were exposed to hydraulic current gradually varying from gentle flow (0.06 m/s) to five times the critical velocity (0.75 m/s) in a 45-min duration test and soil mass loss percentage by the initial dry mass of the treated sample is presented as a measure of soil erodibility in Fig. 7. As expected, with an increase of biocementation cycles, i.e., calcite content, the soil erodibility reduced substantially. The initial dry weight of the samples control, BC1, BC2, and BC3, were measured as 398, 403, 406, and 410 g, respectively. Approximately 7.3% of calcite content resulted in a drastic reduction in erodibility (12% mass loss), while 56% soil mass loss was recorded for control (untreated sand). One biocementation cycle treatment (sample BS3-BC1) produced an average of 2.5% of calcite, reducing the soil loss to 31%. Sample BS3-BC2 with 4.93% calcite content resulted in 22% soil mass loss during the hydraulic flume test. It is worth noting that higher precipitation in the soil pores may hinder the flow of water in the soil matrix and increase the pore water pressure resulting in catastrophic failures. However, the MICP technique is reported to be a great tool to improve soil strength, maintaining an adequate hydraulic conductivity to prevent the build-up of the excess pore water pressure^11^. Theoretically, the percentage pore volume filled with precipitates for samples BS3-BC1, BS3-BC2, and BS3-BC3 considering the observed calcite contents and pore volume (100 ml) is around 3.7, 7.14, and 11.08%. The influence of pore water pressure on erodibility has not been established in the present study, and it certainly is one of the exciting parameters to consider for future studies.

From the visual observation of the soil specimen after the flume test, it was evident that the soil particles start bonding with an increased level of bio cementation. A tough crust was formed on the top of BS3-BC2 and BS3-BC3 the samples, which got eroded with the fluvial current. Insignificant aggregation was observed in the sample BS3-BC1. However, with two and three cycles of biocementation treatment (BS3-BC2 and BS3-BC3), the biocemented soil particles (BCS) were evidently noticed (photos are shown in Supplementary Fig. 4).

Clarà Saracho et al.^27^ addressed the erosion due to tangential flow (similar to river current) by treating the soil specimen with ten pore volume of low concentration of cementation media (0.02 M to 0.1 M) by injection strategy and tested the specimen in the flow velocity ranging from 0.035 to 0.185 m/sec for 120 min in a modified erosion function apparatus (EFA). The study concluded that the treatment with 0.08 M cementation media (calcite content varying from 1.2 to 4%) resulted in negligible erosion in the stated test conditions, and with the increase in MICP treatment, a shift in the erosion mode from particulate mode to block failure was observed indicating that with the increase in calcite content and needle penetration resistance, there might be a threshold for biocemented soil, where the soil erosion might be catastrophic due to block failure. However, in this study, a consistent decrease in soil erodibility is observed with the increase in needle penetration resistance. We found that 7.3% of calcite content was required to control the soil erodibility substantially in the test flow range (0.06 m/s to 0.75 m/s). A similar trend was observed by Kou et al*.*^52^ and Chung et al*.*^53^, where consistent reduction in wave-induced erosion and rainfall-induced erosion was observed with an increase in needle penetration resistance for biocemented fine sand treated with the exogenous bacteria.

Another aspect to note in the context of the applied treatment is the produced ammonia which can be toxic for riparian flora and fauna^15^. From stochiometric calculations, for each biocementation cycle, the produced ammonia is evaluated as 8.5 kg per metric ton of soil treated, and for the best performing treatment approach, i.e., BS3-BC3, the ammonia generated is evaluated as 2.63% by weight of the retained soil. The acceptable limit of ammonia in the surface water was recommended as 17 mg per liter for acute exposure and 1.9 mg/l for chronic exposure for protecting the aquatic life in the freshwater as per the environmental protection agency^54^. With the MICP technique, the threat of produced ammonia crossing the maximum acceptable quantity is highly plausible; however, for the field application, the ammonia generated can be reduced by reducing the quantity of reagents and increasing the period of applications. It is to be noted that the produced ammonia will also be subjected to dilution in the river stream. The average discharge of the Brahmaputra river is around 19.8 megaliters per second in the Assam valley^33^. Therefore, the area of the riverbank to be biocemented must be decided judiciously with the context of the produced ammonia quantity and its possible dilution to non-toxic levels.

#### Microstructure and mineralogical analysis of the biocemented samples

**Figure 8:**
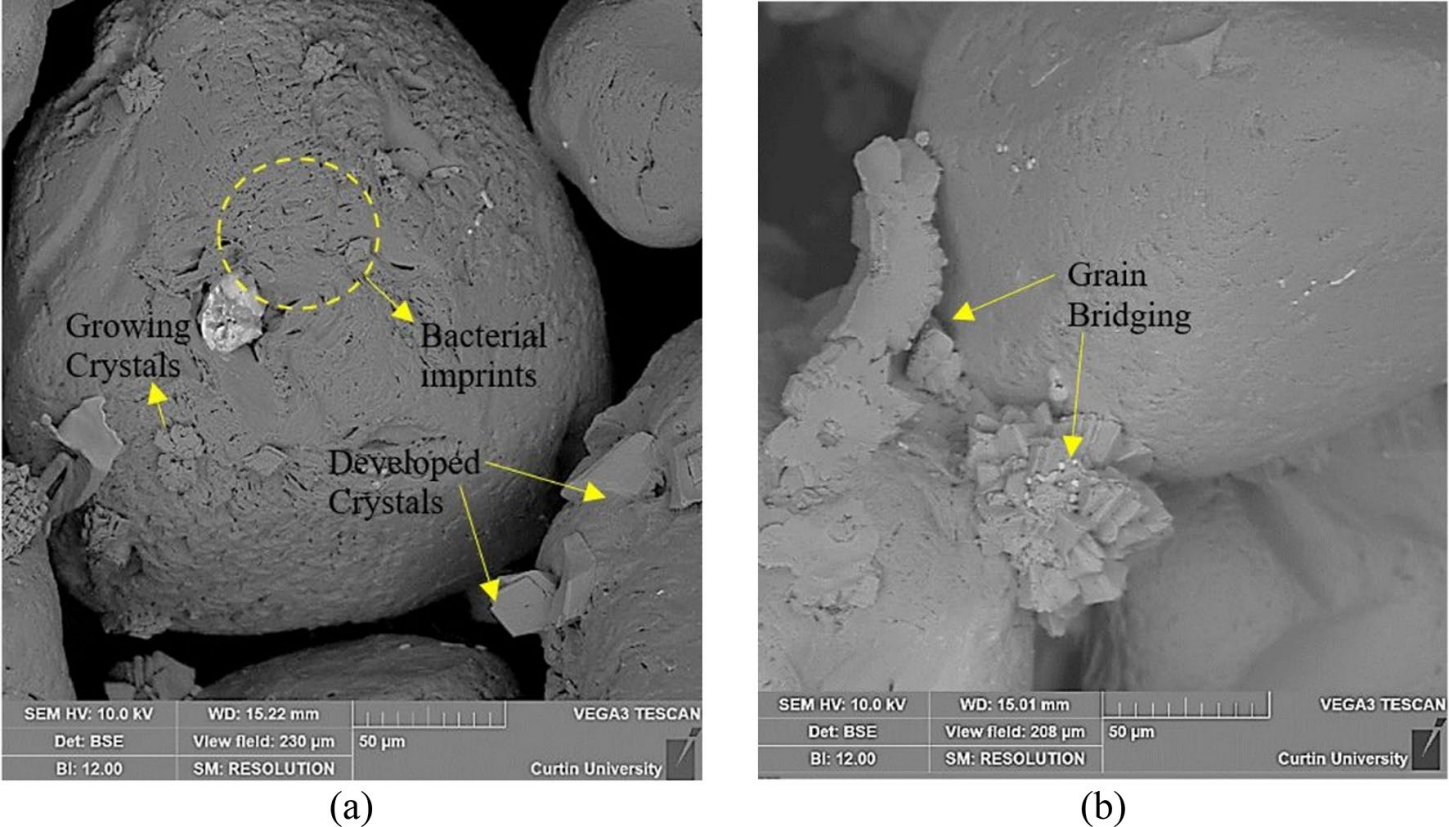
FESEM images of the treated sand grains (**a**). Calcite crystal growing on the sand grains on BS3- BC1 sample (**b**). Calcite bridging in BS3-BC3 samples.

To investigate the influence of different biocementation levels on the erodibility of the treated sand grains, FESEM imaging was conducted for light biocemented samples (BS3-BC1) and heavy biocemented samples (BS3-BC3). While precipitated crystals were observed to be growing on the grooves of sand grains in the light biocemented sample (BS3-BC1), bridging of sand grains with rhombohedral crystals was observed in the heavy biocemented sample (BS3-BC3), as shown in Fig. 8. The effective calcium carbonate bridging between sand grains increases the frictional and cohesive property of sand grains^55,56^, leading to a substantial reduction in the erodibility of the soil. Bacterial imprints were observed in both cases, suggesting the precipitation to be biodgenic^14^. Further EDX analysis on a bridged sand grain (Supplementary Fig. 5) suggested an abundance of silicon and oxygen on the sand grains with a trace amount of chlorine and calcium. This indicates the presence of residual calcium chloride on silica grains. The EDX analysis on the grain bridge indicated the presence of calcium, carbon, and oxygen, suggesting CaCO_3_ precipitation. XRD analysis on treated and untreated sand confirmed the precipitation of calcite. Most of the peaks correspond to quartz (silica). In the biocemented sand sample, a visible peak of calcite was observed at around 29 degrees of 2Ɵ (Details in Supplementary Fig. 6). Therefore, the incorporation of microbial calcite as a binding agent for loose grain silica soil was found to reduce the soil erodibility.

## Conclusions

Biostimulation of ureolytic carbonate precipitating communities from Brahmaputra riverbank soil was successfully conducted in this study. The majority of the isolates belonged to *Sporosarcina* genera highlighting the prevalence of these strains. All the isolated strains were found to have comparable biocementation potential with the conventionally used *Sporosarcina pasteurii* (ATCC 11859) in terms of specific urease activities and quantitative carbonate precipitation potential. This implicates that the enrichment of native bacteria has definite advantages, along with reduced bacterial transport costs and little disturbance to the native microbial communities, flora, and fauna. This study provides promising evidence of the potential of native ureolytic communities from riverbank sites in their ability to mitigate soil erodibility. Further, with needle penetration resistance tests, we were able to quantify the improvement of soil strength with the number of biocementation cycles, i.e., calcite content. However, the distribution of needle penetration resistance was found to be non-uniform, indicating the poor distribution of the precipitated calcite with spraying strategy. The assessment of soil erodibility was conducted in the hydraulic flume, and it was observed that with an increase in the calcite content in treated soil specimens, the eroded soil mass loss decreased substantially. With microstructural analysis (FESEM, EDX, and XRD), the morphology and the mineralogical composition of precipitated crystals were investigated. The calcite crystal growth in the sand grooves and bridging of sand grains was observed as the cause of increased soil strength in the biocemented soil samples. In conclusion, native bacterial communities were found to be effective in mitigating soil erodibility.

In this study, we proposed selective stimulation of the native ureolytic communities for a safe approach to biocementation to control the soil erodibility with minimum intrusion to the native biodiversity of the riverbanks. However, there are numerous concerns for its field application. The alkalinity of growth media and ammonia generation are major concerns for the river ecology and geo-environment. There is a high possibility of ammonia being diluted to negligible levels at the riverbanks; however, this can be threatening to the flora and fauna of the riparian zone. Therefore, a detailed investigation of ammonia dilution or removal strategies will be required, which is not addressed in our study.

As non-uniform soil improvement was observed with spraying, a systematic approach for uniform distribution of calcite with a low concentration of cementation media and continuous application by spraying or injection strategy is recommended. Moreover, it is to be noted that the soil preparation via biocementation and the flume experiments were conducted at room temperature 25 ± 3 °C. The real field conditions in terms of the type of soil (carbon, nitrogen, and soil urease), topography, vegetation cover, and climate may influence the biocementation process, and therefore, these experimental results from the flume tests may not be directly applicable to the field. A detailed investigation of MICP treatment of the riverbank soil based on the real field conditions and durability will be critically useful to design the field application strategy. There are chances of the cementation media getting diluted in the saturated zones of the riverbanks. Therefore, it is essential to consider the application of the MICP for erodibility control only in the low flood seasons to vulnerable riverbanks.

## Methods

### Enrichment of ureolytic communities from the collected soils

Around 10 g of soil from the riverbank and natural slope was procured to the laboratory in sterile containers. One gram of the collected soil was inoculated into the 100 ml enrichment media containing 13 g/l of Nutrient Broth (NB) and 5% Urea in a shaking incubator at 37 °C and 120 rpm for five days. This was followed by two subcultures of the enriched communities into new enrichment media. Confirmation of the ureolytic capability of these communities was verified by inoculating the bacterial culture into urea agar base, wherein the change in color was recorded within 12 h. The details of each of the media (sterile) are reported in Table 2.

**Table 2:** Details of the media used for enrichment, isolation, and cementation.

Media/components	Identifier	Nutrient broth	Urea	Calcium chloride
Enrichment media	NB5U	13 g/l	5%	NA
Growth media	NBU	13 g/l	2%	NA
Precipitation media	PM	1 g/l	2%	50 mM
Fixation media	FM	–	–	25 mM
Cementation media	CM	1 g/l	500 mM	500 mM

#### Isolation, identification, and characterization of the isolates

After the enrichment of ureolytic communities, we attempted to isolate the efficient ureolytic cultures from the consortia. For this, the enriched consortia were serially diluted and inoculated on NBU agar plates and single colonies were obtained. These colonies were then screened for qualitative urease production on urea agar plates. Six highly ureolytic cultures were screened. This was followed by the identification of these cultures via 16S rRNA genomic sequencing. The 16S rRNA sequencing was conducted at NCMR (National Centre for Microbial Research, India). The sequences of the isolated strains were analyzed and compared with the highly similar sequences available on the NCBI database utilizing nucleotide BLAST. The DNA sequences of the isolated strains were aligned with the reference sequences of the obtained highly similar strains by the MUSCLE algorithm and the phylogenetic tree was constructed by the neighbor-joining method using Mega-X software^57^.

The screened isolates were further characterized based on their biochemical properties, growth characteristics, urease activity, and calcium utilization potential. The biochemical properties of the isolates were evaluated using the Microbact 12 A, 12B, and 24 E kit. The isolates were grown in the NBU media for four days at 37 °C and 180 rpm in a shaking incubator to evaluate growth characteristics and urease activity. The performance of isolates was compared with the comprehensively used SP (*Sporosarcina pasteurii* ATCC 11859) and the artificial consortia. The artificial consortia were created by mixing an equal proportion of the isolated strains from site 1.

The urease activity (mM urea hydrolyzed per hour) of the bacterial isolates was evaluated following the electrical conductivity method^58^. For assay of urease activity, 1 ml of bacteria is mixed with 9 ml of 1.11 M urea, and the change in electrical conductivity was measured for 5 min at room temperature (25 ± 3 °C). The variation in electrical conductivity rate (mS per cm per minute) is measured and the urease activity is calculated taking the dilution factor into account. The ratio of the urease activity and the bacterial optical density (OD_600_) is defined as the specific urease activity (mM urea hydrolyzed h^−1^ OD_600_^–1^).

### Analytical methods for evaluation of biocementation potential of the isolates

The biocementation potential of the efficient bacterial isolate was evaluated following a modified method from Dhami et al.^46,47^. 1% overnight grown bacterial culture (OD_600_ = 1) was added in the precipitation media (PM) consisting of 1 g/l Nutrient Broth, 2% urea, and 50 mM of CaCl_2_ in a shaking incubator at 37 °C and 120 rpm. A negative control set consisting of only cementation media was also observed for the test duration to check the possibility of abiotic precipitation.

The soluble calcium depletion was evaluated by measuring soluble Ca^2+^ for 48 h by the EDTA titration method^14^. For evaluation of soluble Ca^2+^, 10 ml of the inoculated cementation media was taken out at different time intervals and centrifuged at 4 °C and 8000 rpm for 5 min. The supernatant was supplemented with 0.5 ml of 5 N sodium hydroxide and few drops of hydroxy-naphthol blue indicator, and the mixture was titrated against the 250 mM EDTA. The required EDTA to change color from pink to blue was noted and compared with a standard calibration plot of CaCl_2_ solutions ( ranging 5–100 mM. The performance of isolates was compared with comprehensively used SP (*Sporosarcina pasteurii* ATCC 11859) and the consortium. A negative control set consisting of only precipitation media was also observed for the test duration to check the possibility of abiotic precipitation.

At the end of four days, the precipitates were collected on Whatman no. 1 filter paper and washed with sterile water as per the protocol of Dhami et al.^46^. The precipitates were air-dried after filtration at room temperature for 48 h, weighed, and analyzed for gravimetric, microstructural, and mineralogical analysis.

### Soil sample preparation

Fine sand equivalent to Brahmaputra riverbank sand was procured from Cook Industrial Minerals, Western Australia. The sand was filled at a 40% relative density of 1.6 g/cc in the containers. For the needle penetration test and calcite content measurements, the soil samples were prepared in Petri dishes of diameter 5 cm and depth 1 cm. After the preliminary investigation on the penetration resistance improvement, samples for the hydraulic flume tests were prepared in a container of length 9.25 cm, width 5.8 cm, and depth 4 cm. Separate samples were made for flume test and needle penetration test as the Needle penetration prior to hydraulic flume test can damage the bio cemented crust formed on the top surface. The soil specimens were treated with 0.5 M of equimolar cementation media, as suggested by Jiang and Soga^45^ and Porter et al*.*^55^. The spraying strategy for the treatment was considered as it is a convenient strategy for the field application. As per the recommendation of Wang et al*.*^25^, non-uniform calcite precipitation was observed with surficial percolation strategy and spraying strategies were suggested as a viable alternative. For uniform precipitation, the biocementation solutions were supplied in three steps, which include the application of fixation solution, ureolytic bacteria solution, and cementation solution, as suggested by Harkes et al*.*^20^. In the current study, one biocementation cycle (BC1) is completed by spraying one pore volume of fixation solution, one pore volume of bacterial solution (at optical density ≥ 1), and one pore volume of the cementation solution (two times) consecutively after the retention period of 24 h for each step. Similarly, samples with two and three biocementation cycles (BC2 and BC3) treatments were prepared to investigate improved soil properties. Details of all the samples prepared for experiments have been summarized in Table 3.

**Table 3:** Details of soil samples prepared.

Sample identifier	Bacteria	Biocementation treatment cycles
SP-BC1	Sporosarcina pasteurii	1
Con-BC1	Consortia	1
BS3-BC1	Isolate BS3	1
BS3-BC2	Isolate BS3	2
BS3-BC3	Isolate BS3	3
Control	No bacteria	–

#### Needle penetration test

Needle penetration test was performed at a penetration rate of 15 mm/minute for 5 mm depth, and the ratio of force to depth (N/mm) has been reported as needle penetration index (NPI) as recommended by ISRM (International Society of Rock Mechanics)^49^. The NPI values were mapped for 25 points. The Chenille needle #22, with a diameter of 0.86 mm, was mounted on a universal testing machine (Shimadzu AGS-X). First, the average needle penetration indices of samples SP-BC1, BS3-BC1, and Consortia-BC1 were compared to investigate the influence of different strains and the same level of biocementation on soil strength improvement. Later, to quantify the improvement in soil strength, needle penetration resistance was investigated for samples treated with native bacteria-based two and three biocementation cycles (BS3-BC2 and BS3-BC3).

#### Erodibility test for a bed slope in hydraulic flume

The sample treated with the most suitable native strain at a different level of biocementation (BS3-BC1, BS3BC2, and BS3-BC3) were further tested and compared with the untreated sand on the hydraulic flume-based erodibility test. The erodibility of soil was evaluated in a 12 m long, Armfield Engineering ltd. S5 tilting flume with the glass walls 320 mm high and 300 mm wide (internal breadth). The treated soil containers were plugged into an acrylic bed slope in the flume (Supplementary Fig. 2). The samples were saturated and then the flow velocity was increased stepwise at intervals of a 5-min time interval (Supplementary Fig. 3). This stepwise incremental flow methodology was considered based on the study by Clarà Saracho et al*.*^27^, as the critical flow velocity is expected to increase with the biocementation treatment levels. The flow was operated using the control panel and velocity was measured on three different points near the sample using a pulse velocity meter. The critical flow velocity for the riverbank soil calculated from Briaud’ (2008) suggested equation^59^ was found to be 0.15 m/s.

3 $$V_{C} = 0.35\left( {D_{50} } \right)^{0.45}$$

here* V*_*c*_ is the critical flow velocity required to initiate the detachment of soil particles, and D_50_ is the mean diameter of the cohesionless soil. The samples were installed 6 m downstream in the hydraulic flume.

#### Microstructural and Mineralogical analysis of the treated soil

The ZEISS Neon 40 EsB dual FESEM/FIBSEM instrument equipped with Field Emission Scanning Electron Microscopy (FESEM) and Energy-dispersive X-ray spectroscopy (EDX) was used for the microstructural investigation. The air-dried treated soil specimens were mounted on the aluminum stub for its microstructural and elemental composition analysis. The samples were coated with a 10 nm platinum coating. For mineralogical analysis of the precipitates, all the samples were micro-ionized (particle size < 10 microns) and analyzed by X-ray diffraction (XRD) on Bruker D8 advanced diffractometer with Nickel filtered Cu-Kα radiation varying 2θ from 5°–100° with a step size of 0.013°. The phase identification was made utilizing COD and ICDD databases with Bruker EVA.

The calcite content of the treated soil was measured by the acid washing method. 5-g treated soil was collected in triplicates from the crust after the flume test and washed with 20 ml of 1 M HCl. The calcite content was determined based on the gravimetric difference between the collected soil sample (5 g) and retained weight of the acid-washed soil on Whatman filter grade 1^60^.

### Statistical analysis

All samples were analyzed in triplicates, and the average was compared except for the needle penetration test and flume test. The data were analyzed by one-way analysis of variance (ANOVA). For the needle penetration test, the tests were performed at 25 points on the crust and the average value with the standard deviation is reported.

## Supplementary Information

Supplementary Information.
