# Author Correction: Biocementation mediated by native microbes from Brahmaputra riverbank for mitigation of soil erodibility

**DOI:** 10.1038/s41598-021-97283-7

**Published:** 2021-09-01

**Authors:** Anant Aishwarya Dubey, K. Ravi, Abhijit Mukherjee, Lingaraj Sahoo, Moses Akindele Abiala, Navdeep K. Dhami

**Affiliations:** 1 Indian Institute of Technology, Guwahati, 781039 India; 2 Curtin University, Perth, WA 6152 Australia; 3 Mountain Top University, Prayer City, Nigeria

Correction to: *Scientific Reports* 10.1038/s41598-021-94614-6, published online 27 July 2021

The original version of this Article contained an error in Figure 4, where Figure 4d was a duplication of Figure 4e. The original Figure 4 and accompanying legend appear below.

In addition, in Table 1, in the column “Properties”,

“Clay content % (≤ 0.075 mm)”.

now reads:

“Clay content % (≤ 0.002 mm)”.

The original Article has been corrected.

**Figure 4:**
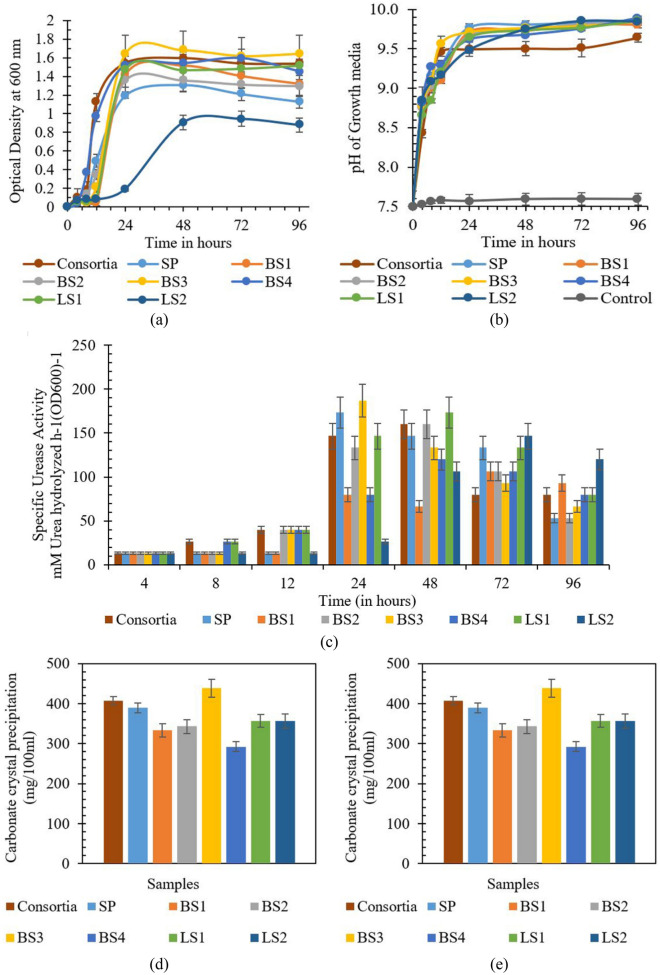
(**a**) Growth characteristics, (**b**) pH, (**c**) specific urease activity, (**d**) calcium utilization rate, and (**e**) carbonate precipitation rate of the isolates and consortia.

