# Supplementary material for: Biocementation mediated by native microbes from Brahmaputra riverbank for mitigation of soil erodibility
Source: Sci Rep. 2021 Jul 27;11:15250. doi: 10.1038/s41598-021-94614-6 (PMC8316328; doi:10.1038/s41598-021-94614-6)
Supplement: Supplementary file 1 — Supplementary Information. [file 41598_2021_94614_MOESM1_ESM.docx]

# Biocementation mediated by stimulated ureolytic microbes from Brahmaputra riverbank for mitigation of soil erosion

Anant Aishwarya Dubey^1,2^, K. Ravi^1^, Abhijit Mukherjee^2^, Lingaraj Sahoo^1^, Abiala Moses Akindele^3^, Navdeep K Dhami^2*,^

^1^Indian Institute of Technology, Guwahati, India-781039.

^2^Curtin University, Perth, Western Australia, Australia-6152

^3^Mountain Top University, Nigeria

[^2*^navdeep.dhami@curtin.edu.au](mailto:2*navdeep.dhami@curtin.edu.au)

**Supplementary data**

Captions of figures

Supplementary Figure 1. (a). EDX and (b). XRD analysis of the crystal precipitates from BS3

Supplementary Figure 2. Photograph and plan of the sample setup in the Armstrong tiling hydraulic flume

Supplementary Figure 3. Flow program for the bed erosion test in the flume

Supplementary Figure 4. Before and after flume erosion test images of the soil samples

Supplementary Figure 5. FESEM-EDX layered image of elements of treated sand grains (BS3-BC3) confirming the presence of Calcite bridging over sand grains

Supplementary Figure 6. XRD plot of the sand (untreated) and bio cemented sand samples

| 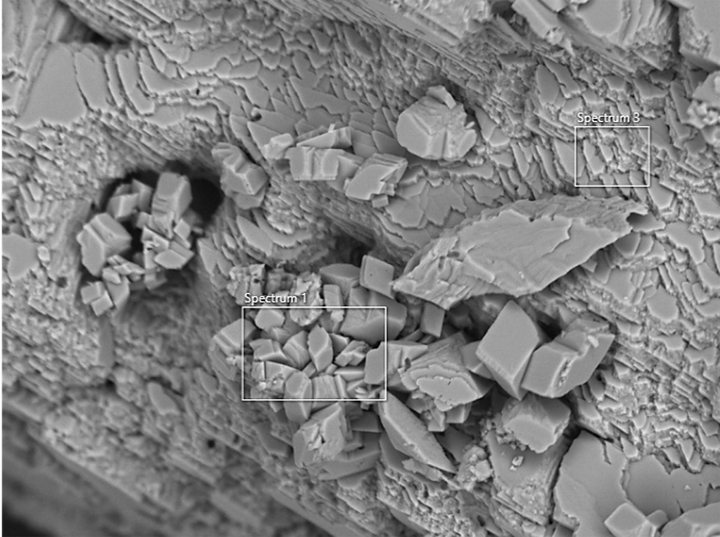 | 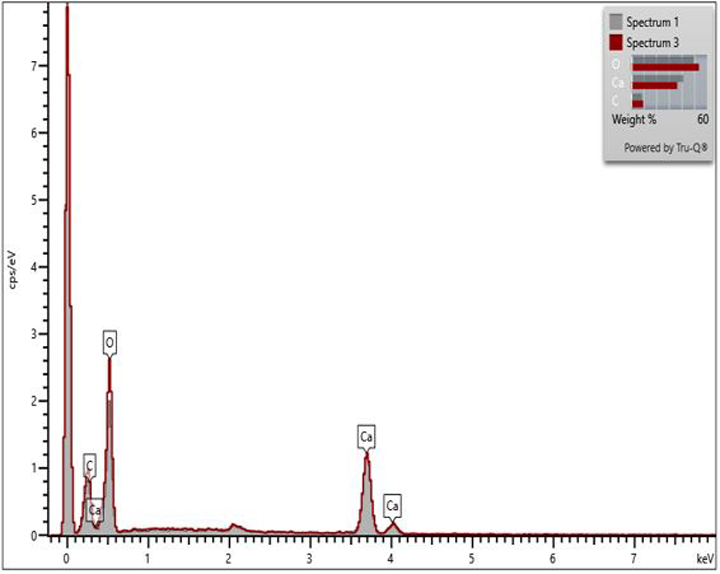 |
| --- | --- |
| 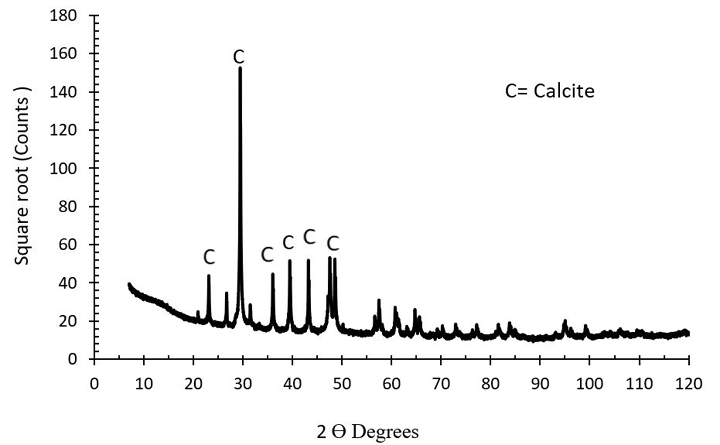 | |

Supplementary Figure 1 (a). EDX and (b). XRD analysis of the crystal precipitates from BS3


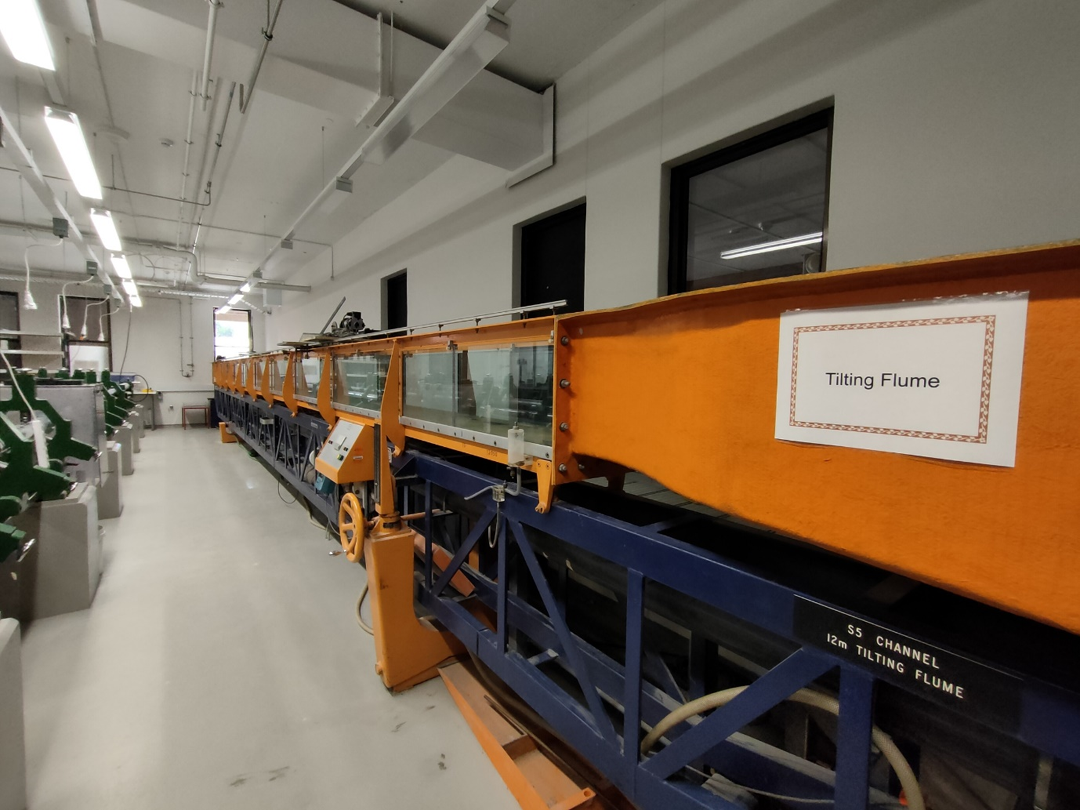


(a)


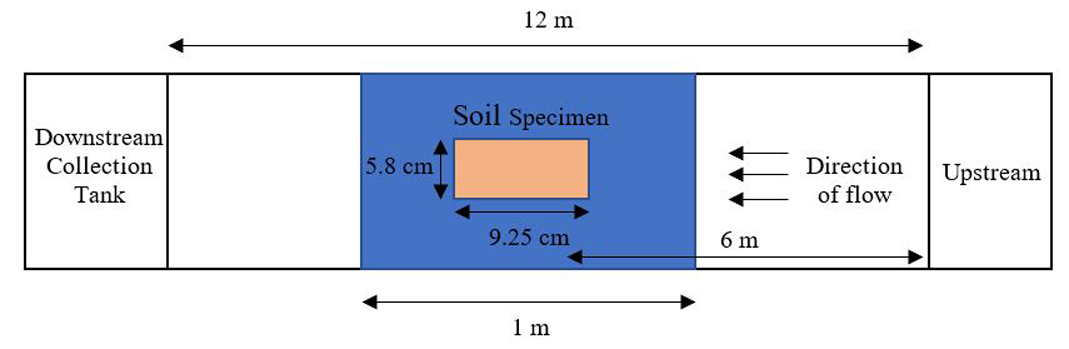


(b)

Supplementary Figure 2 Photograph and plan of the sample setup in the Armstrong tiling hydraulic flume


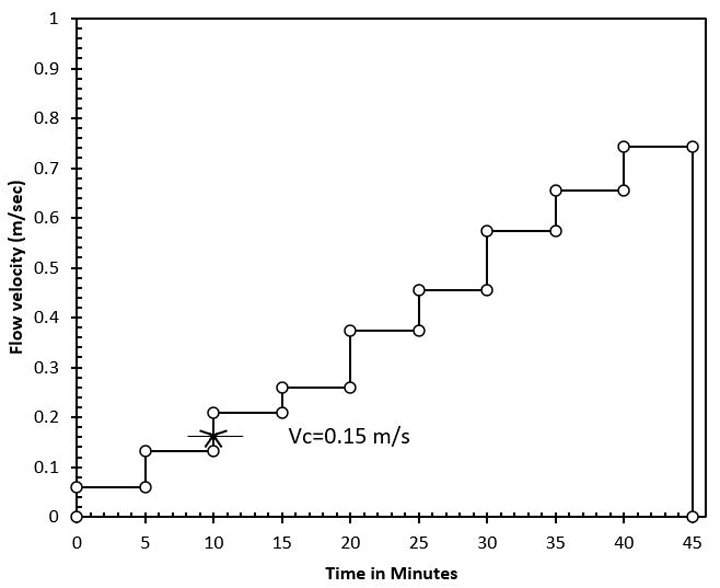


Supplementary Figure 3 Flow program for the bed erosion test in the flume


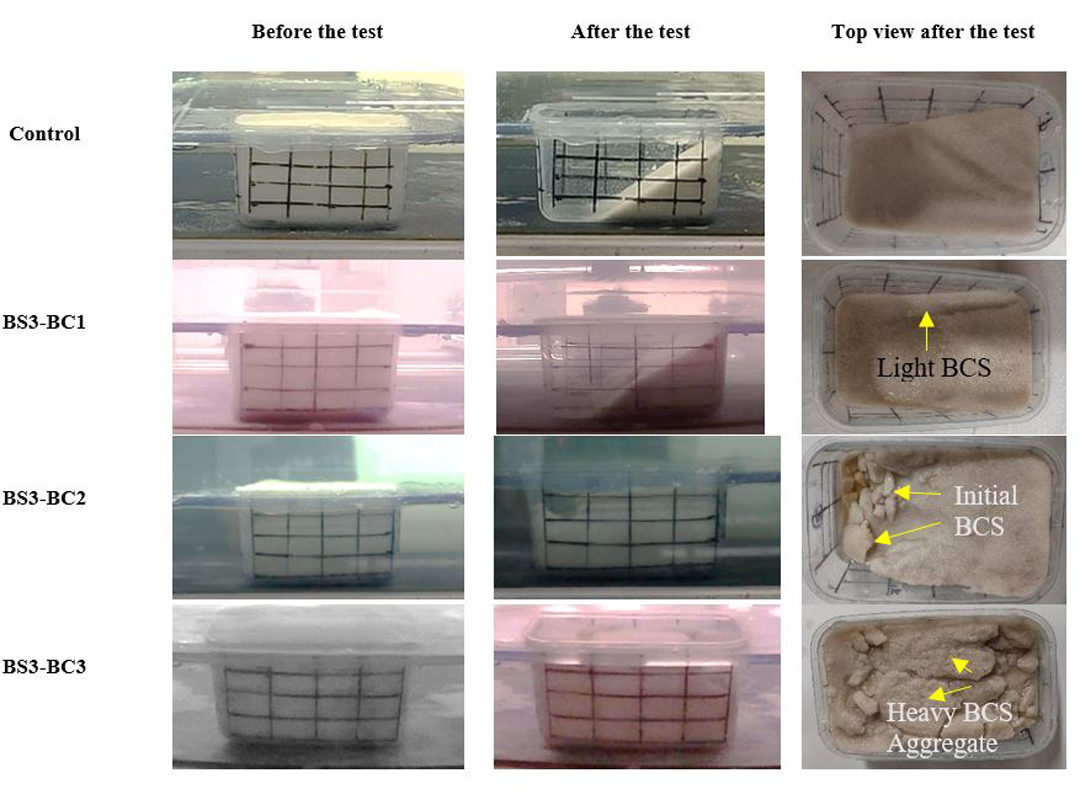


Supplementary Figure 4 Before and after flume erosion test images of the soil samples


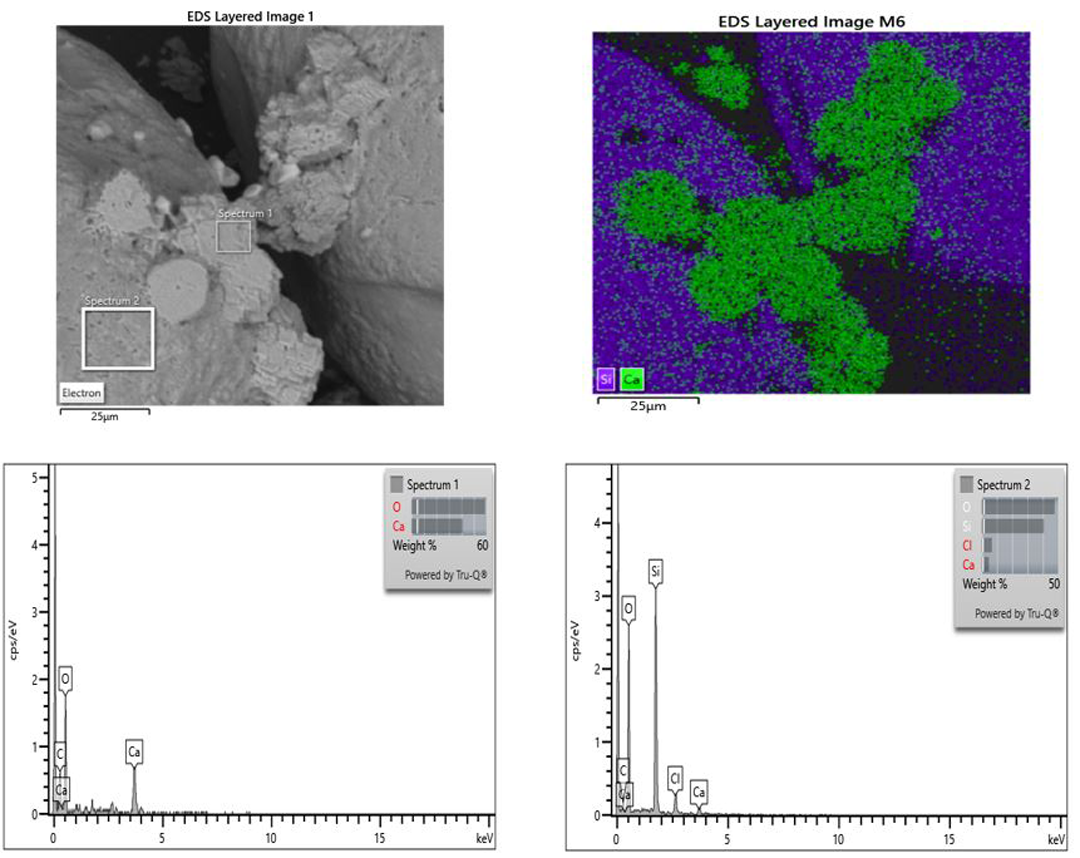


Supplementary Figure 5 FESEM-EDX layered image of elements of treated sand grains (BS3-BC3) confirming the presence of Calcite bridging over sand grains


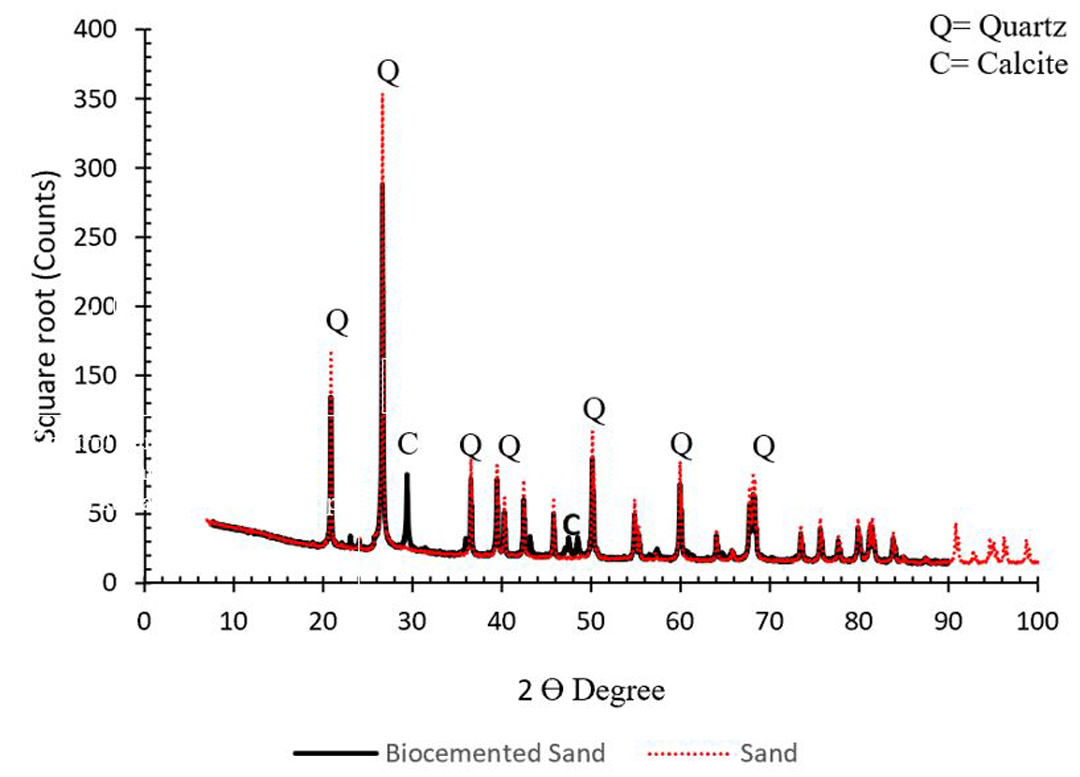


1

2

3

4

1. Control Panel

2. Flow Channel

3. Water storage tank and pump

4. Tailgate

Supplementary Figure 6 XRD plot of the sand (untreated) and bio cemented sand samples

**Captions of table**

Supplementary Table 1. Details of isolated strains

Supplementary Table 2. Comparison of the Physicochemical properties of the isolates with respect to *Sporosarcina pasteurii* (ATCC 11859)

Supplementary Table 1. Details of isolated strains

| S.N. | Isolate  Name | Experimental ID | Site | Closest relative | Similarity (%)  by NCMR | NCBI accession number |
| --- | --- | --- | --- | --- | --- | --- |
| 1 | BS3B3PP | BS1 | Site 1 | *Sporosarcina pasteurii* NCIMB 8841(T) | 98.85 | MW024144 |
| 2 | BS3C3 | BS2 | Site 1 | *Sporosarcina pasteurii* NCIMB 8841(T) | 97.96 | MW024145 |
| 3 | BRS2A3 | BS3 | Site 1 | *Sporosarcina pasteurii* NCIMB 8841(T) | 98.00 | MW024146 |
| 4 | BRS2B3 | BS4 | Site 1 | *Sporosarcina pasteurii* NCIMB 8841(T) | 98.41 | MW024147 |
| 5 | LS3PPA | LS1 | Site 2 | *Pseudogracilibacillus auburnensis* P-207(T) | 97.29 | MW024148 |
| 6 | LS2PPA | LS2 | Site 2 | *Sporosarcina pasteurii* NCIMB 8841(T) | 98.31 | MW024149 |

Supplementary Table 2: Comparison of the Physicochemical properties of the isolates with respect to *Sporosarcina pasteurii* (SP)

| **Sample** | **Endospore Staining** | **Gram Staining** | **Oxidase Test** | **Lysine** | **Ornithine** | **H2S** | **Glucose** | **Mannitol** | **Xylose** | **ONPG** | **Indole** | **Urease** | **VP** | **Citrate** | **TDA** | **Gelatin** | **Malonate** | **Inositol** | **Sorbitol** | **Rhamose** | **Sucrose** | **Arabinose** | **Adonitiol** | **Raffinose** | **Salicin** | **Arginine** |
| --- | --- | --- | --- | --- | --- | --- | --- | --- | --- | --- | --- | --- | --- | --- | --- | --- | --- | --- | --- | --- | --- | --- | --- | --- | --- | --- |
| **SP** | 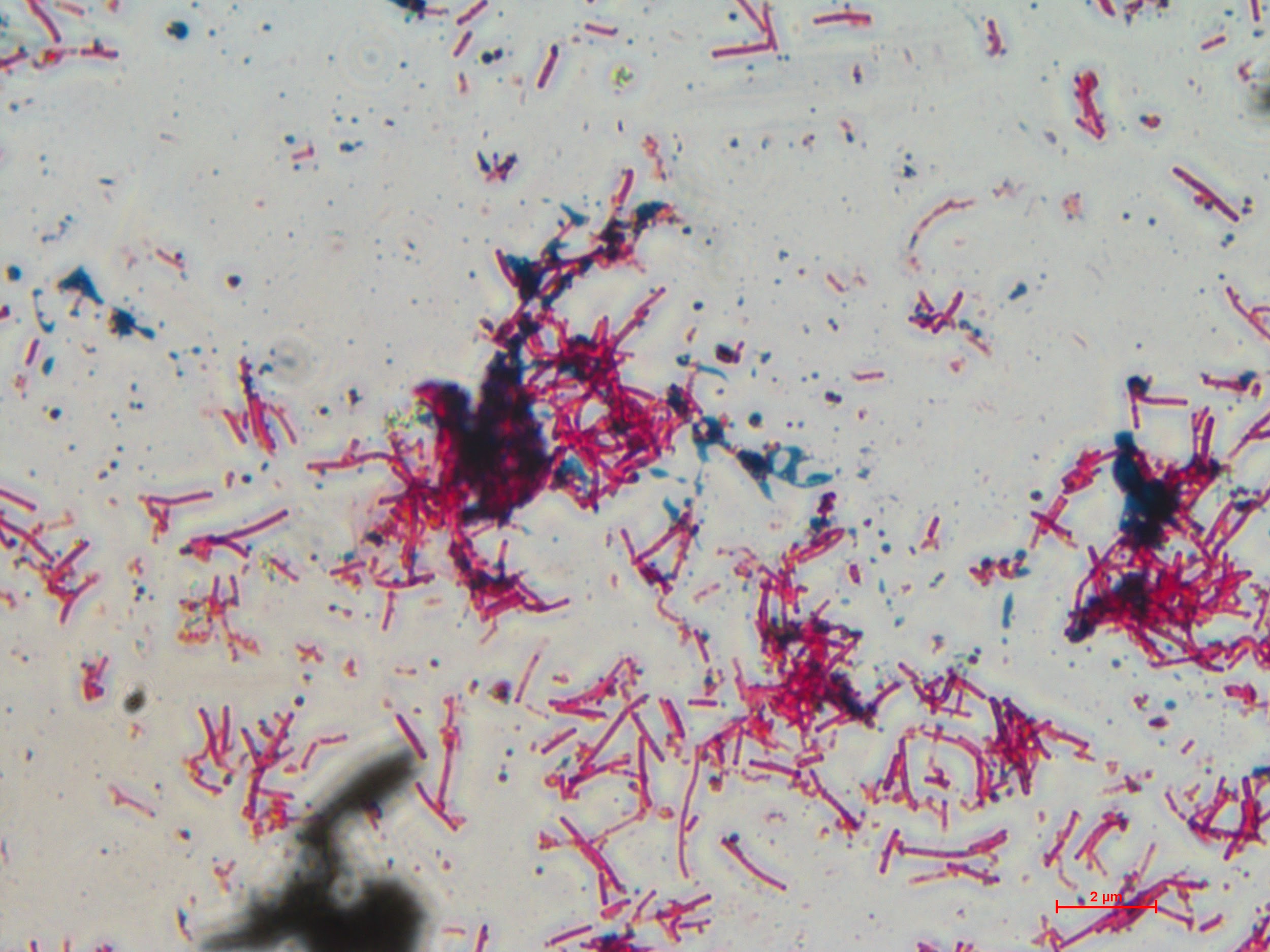 | 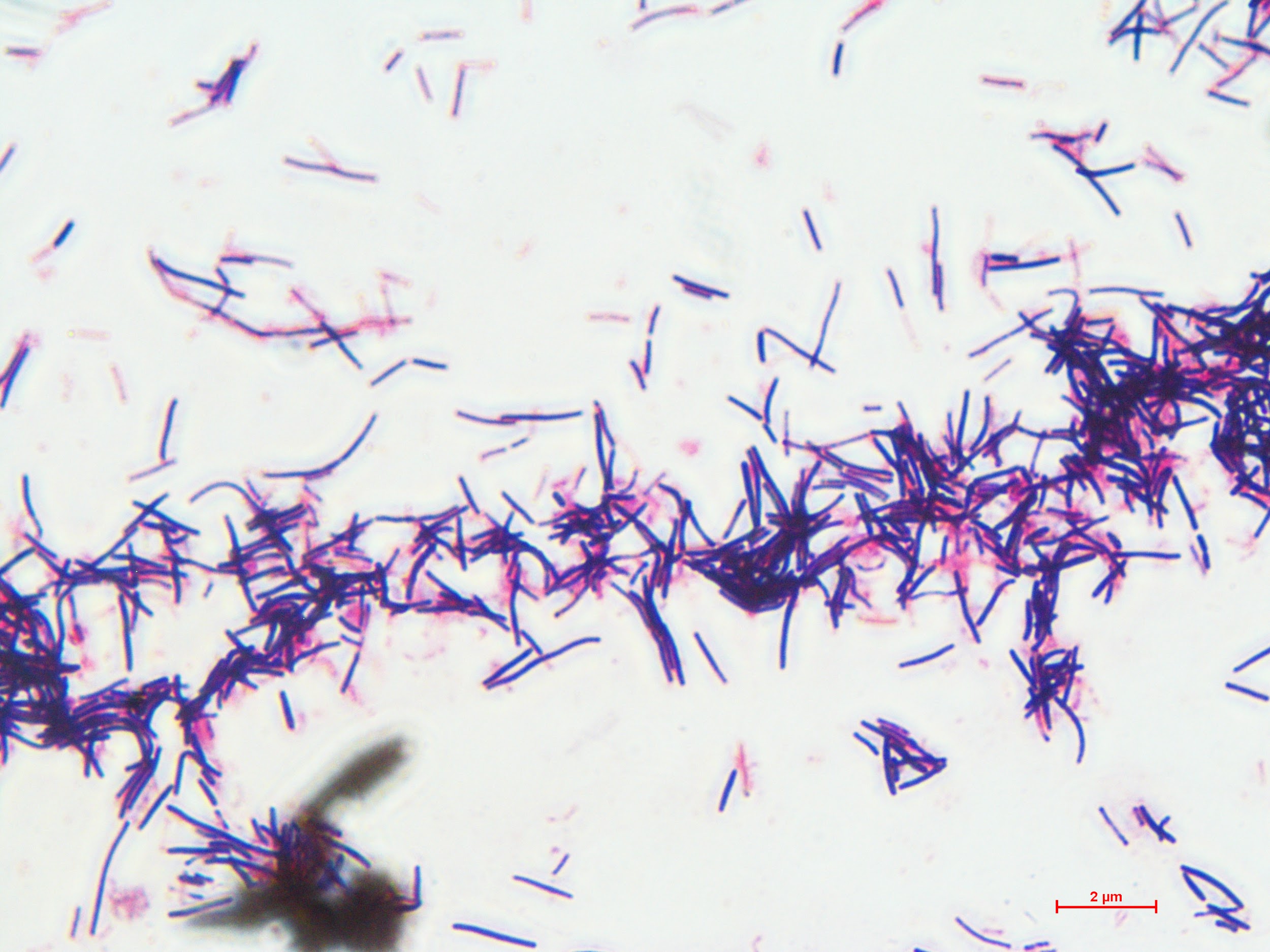 | P | **P** | N | N | N | N | N | **P** | N | P | P | N | P | N | N | N | N | N | N | N | N | N | N | N |
| **BS1** | 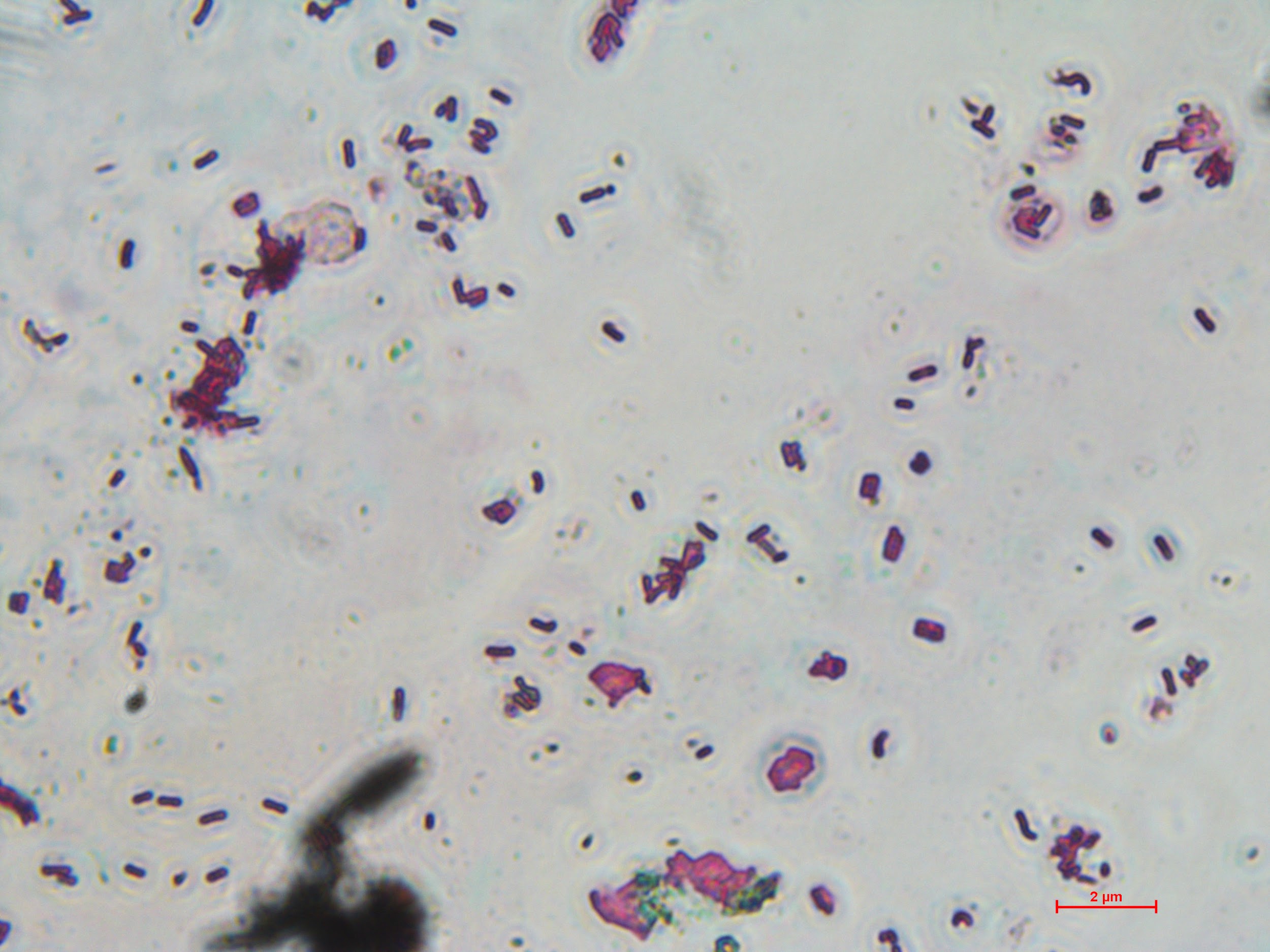 | 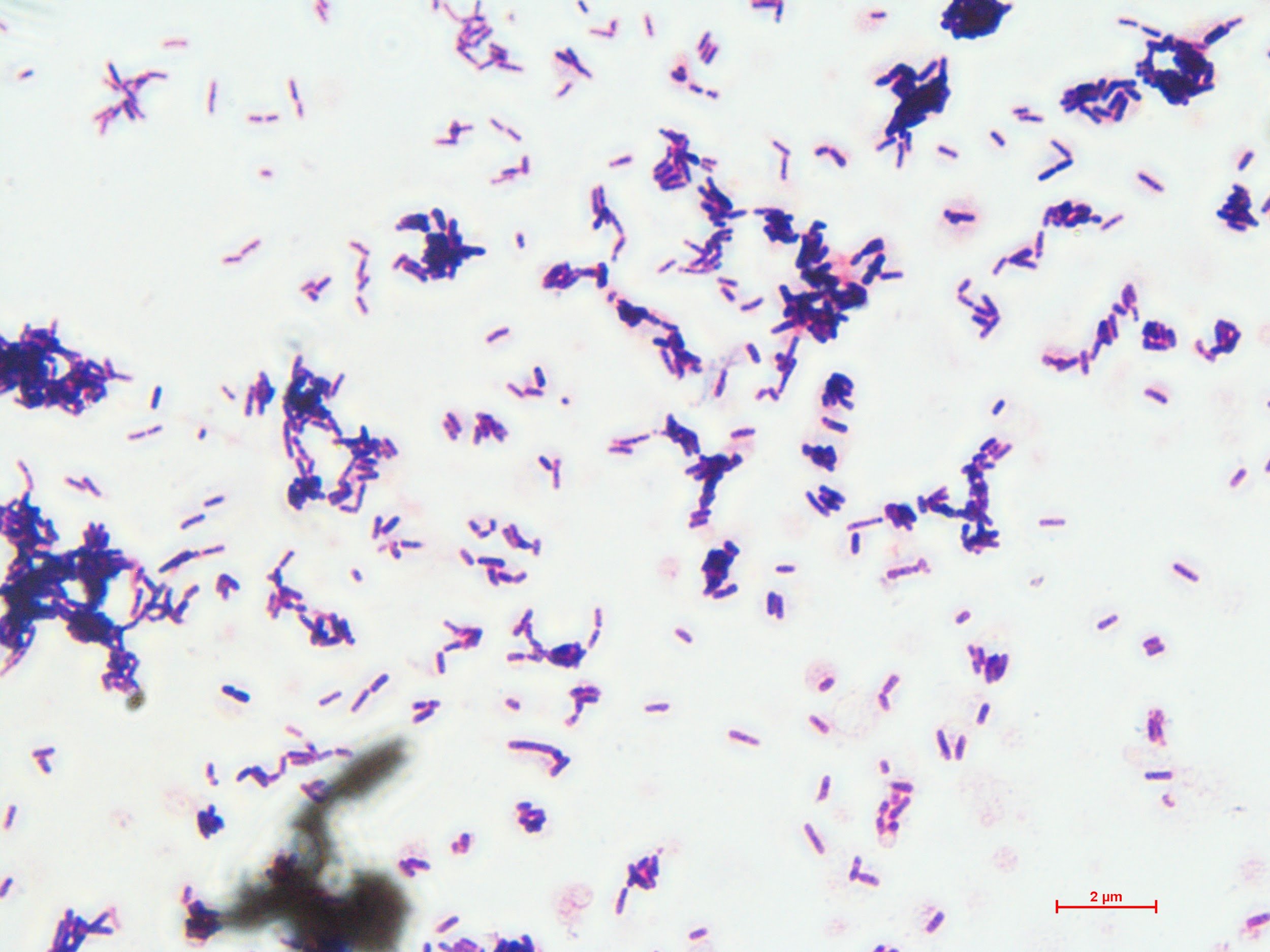 | P | **N** | N | N | N | N | N | **N** | N | P | P | N | P | N | N | N | N | N | N | N | N | N | N | N |
| **BS2** | 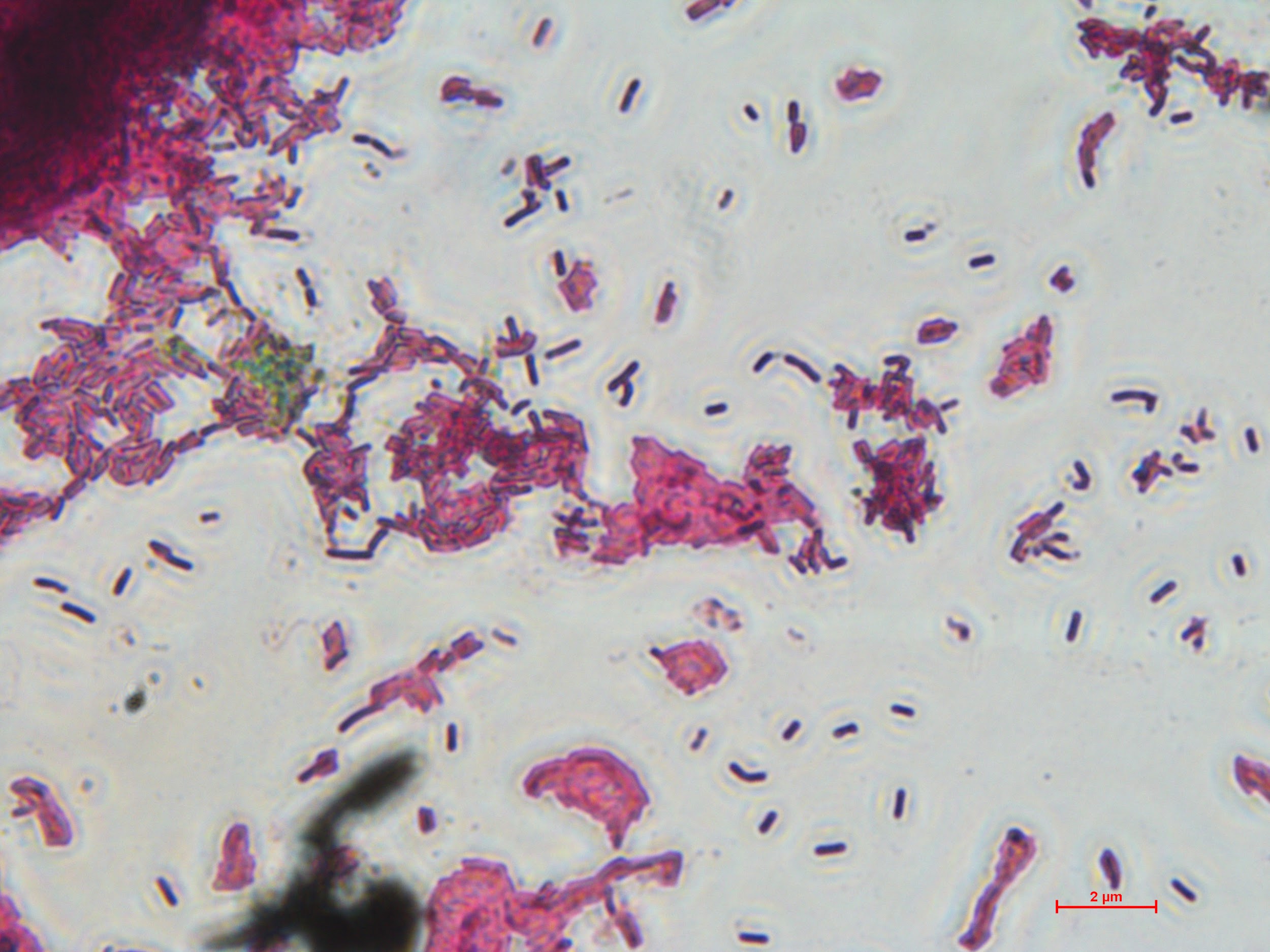 | 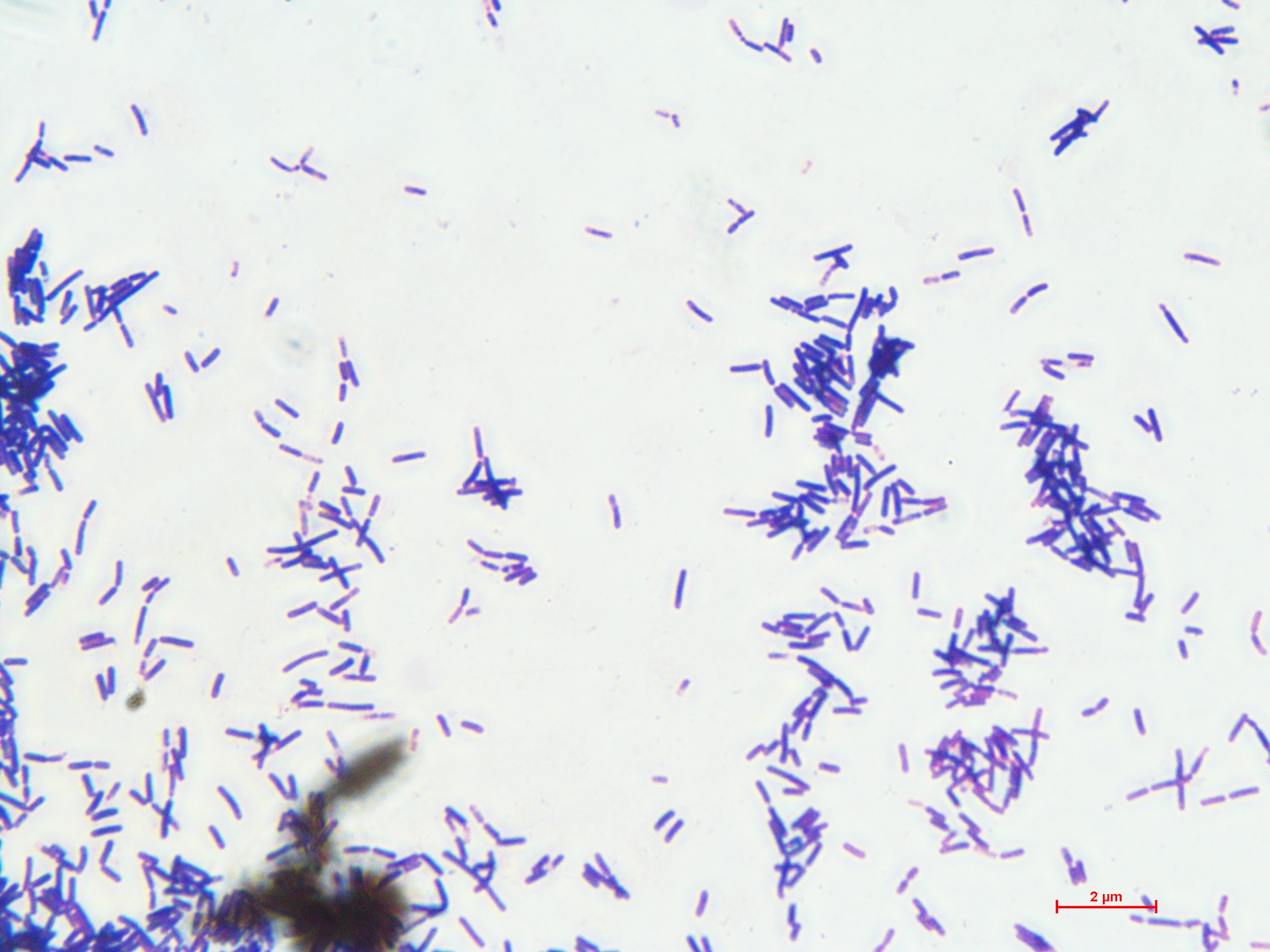 | P | **N** | N | N | N | N | N | **N** | N | P | P | N | P | N | N | N | N | N | N | N | N | N | N | N |
| **BS3** | 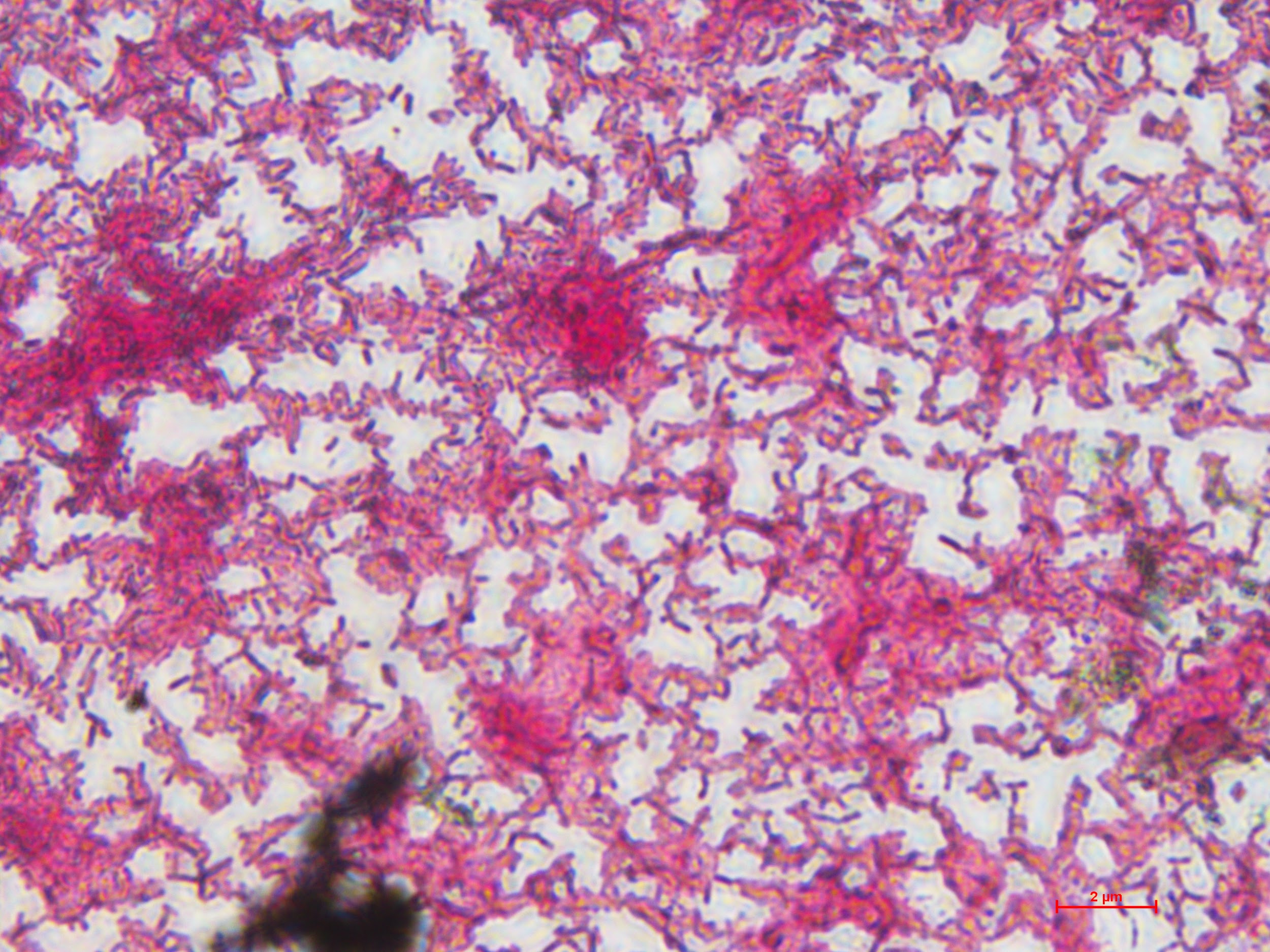 | 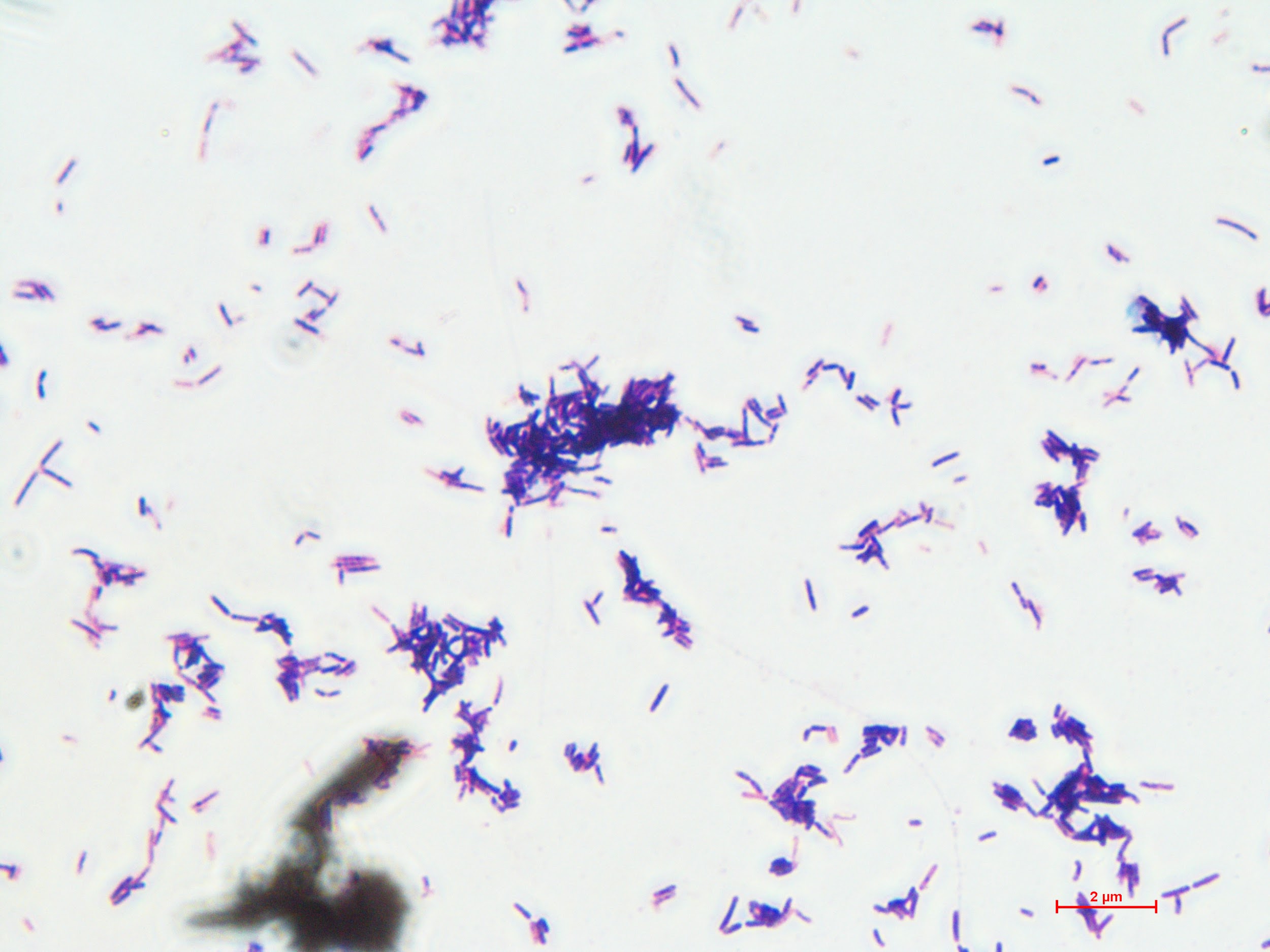 | P | **N** | N | N | N | N | N | **N** | N | P | P | N | P | N | N | N | N | N | N | N | N | N | N | N |
| **BS4** | 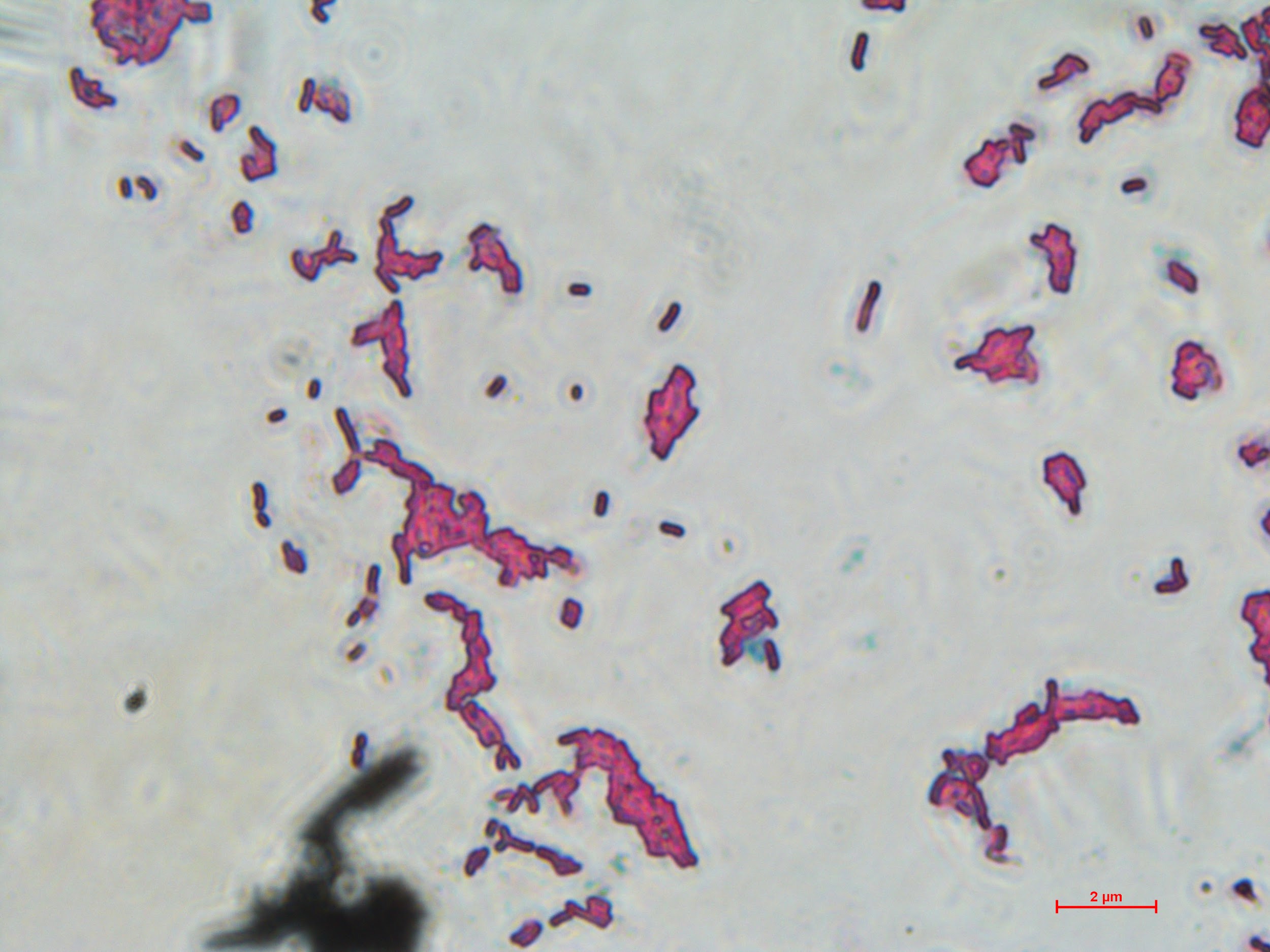 | 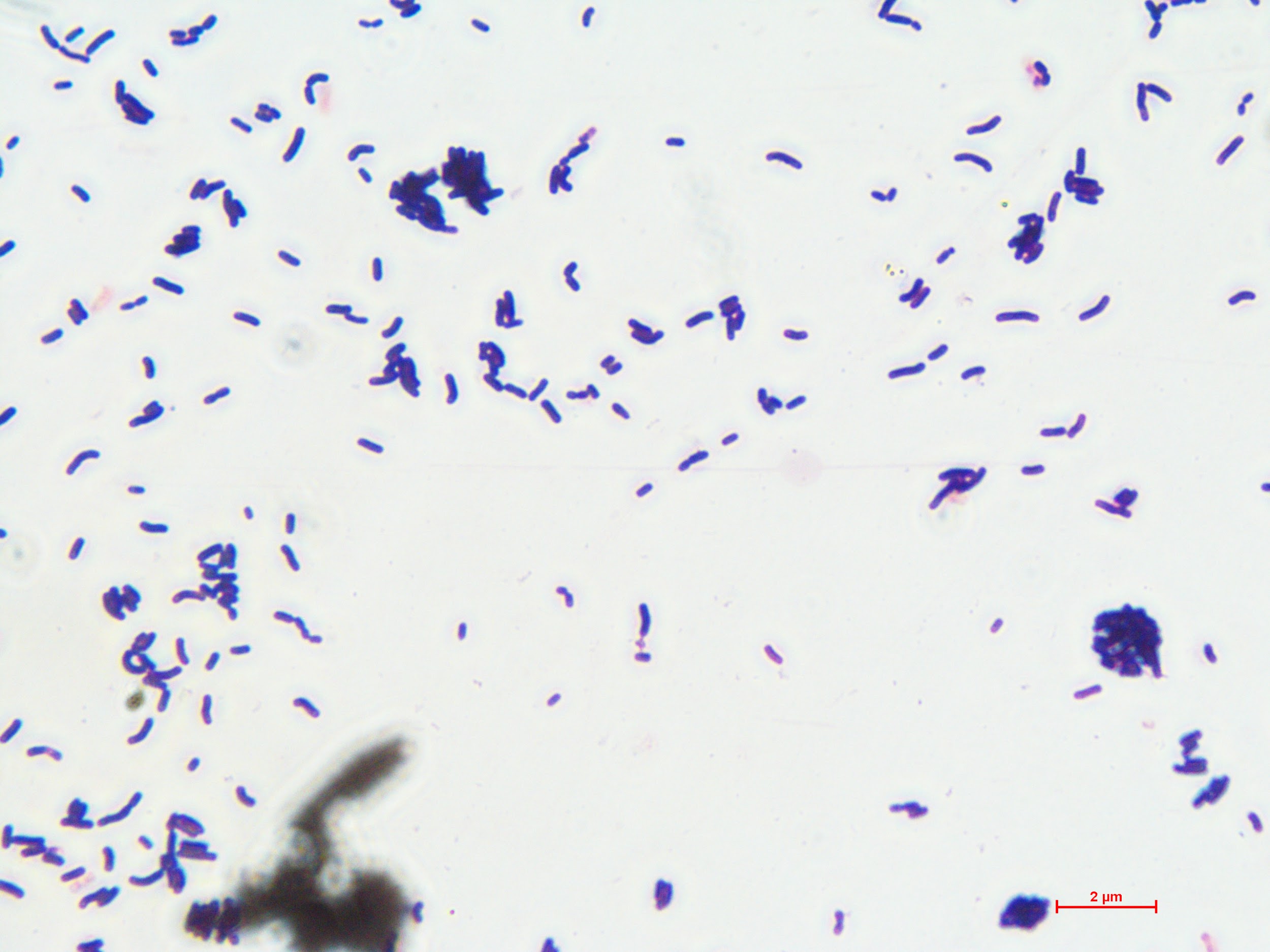 | P | **N** | N | N | N | N | N | **N** | N | P | P | N | P | N | N | N | N | N | N | N | N | N | N | N |
| **LS1** | 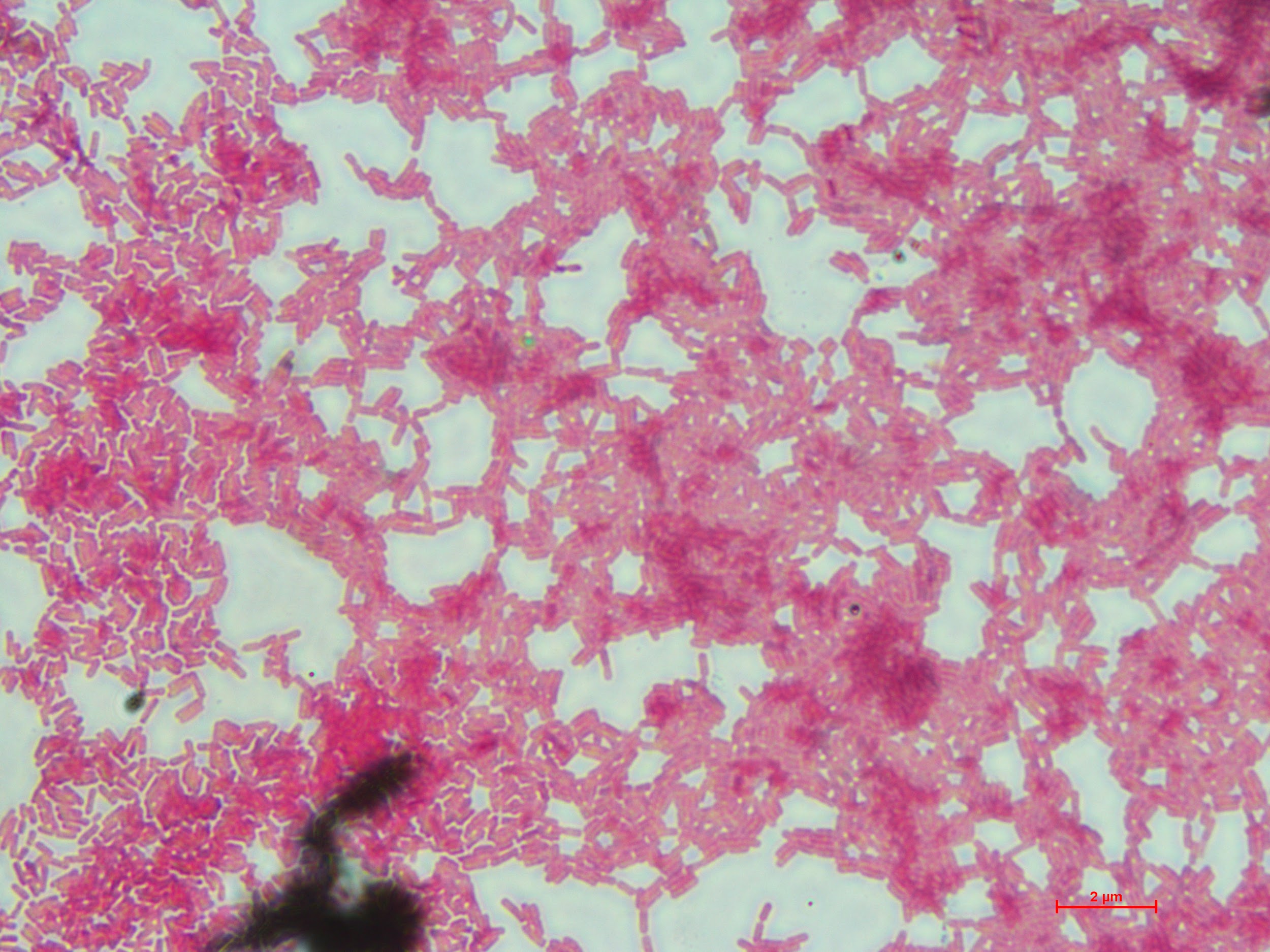 | 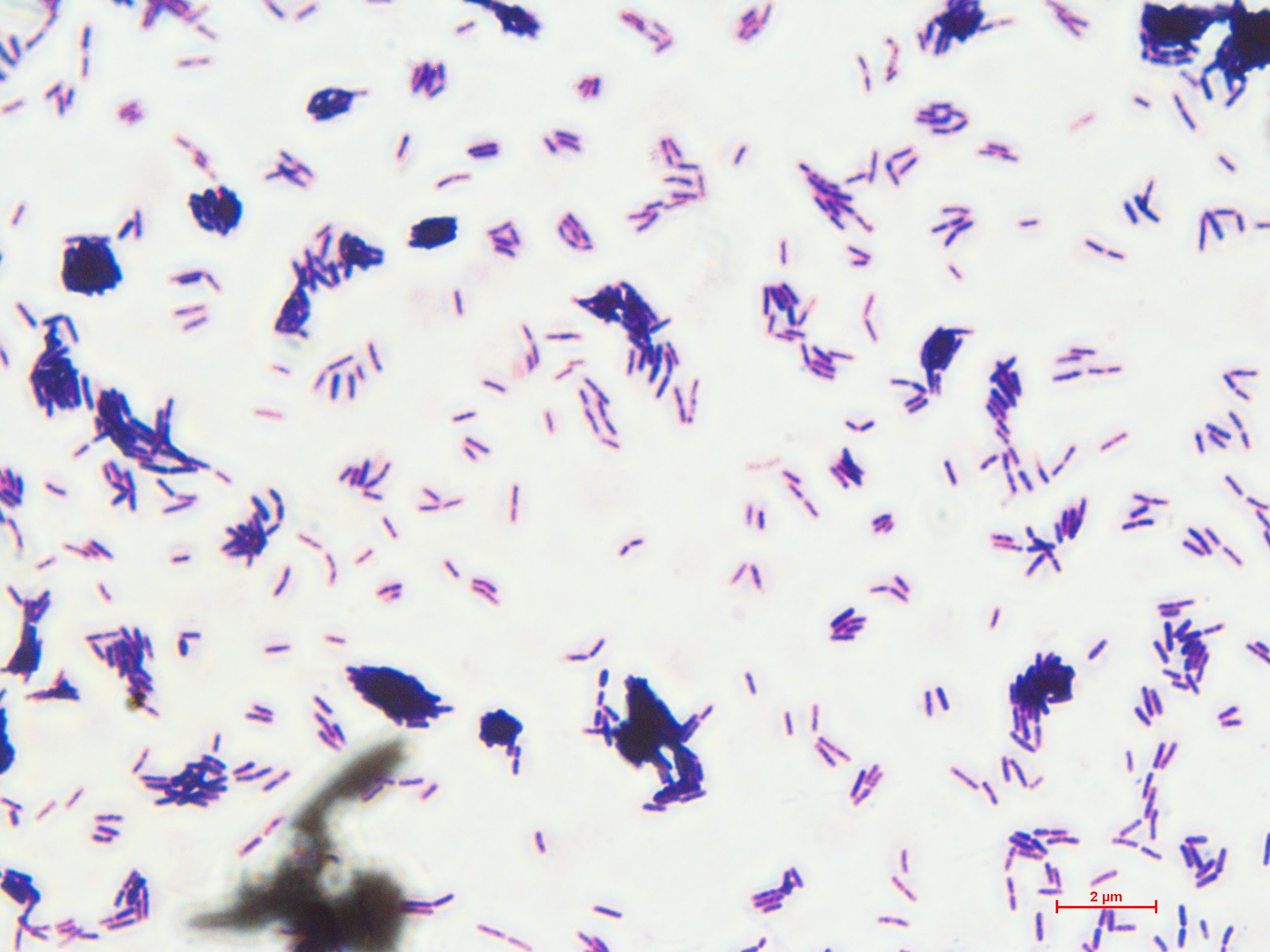 | P | **N** | N | N | N | N | N | **N** | N | P | P | N | P | N | N | N | N | N | N | N | N | N | N | N |
| **Ls2** | 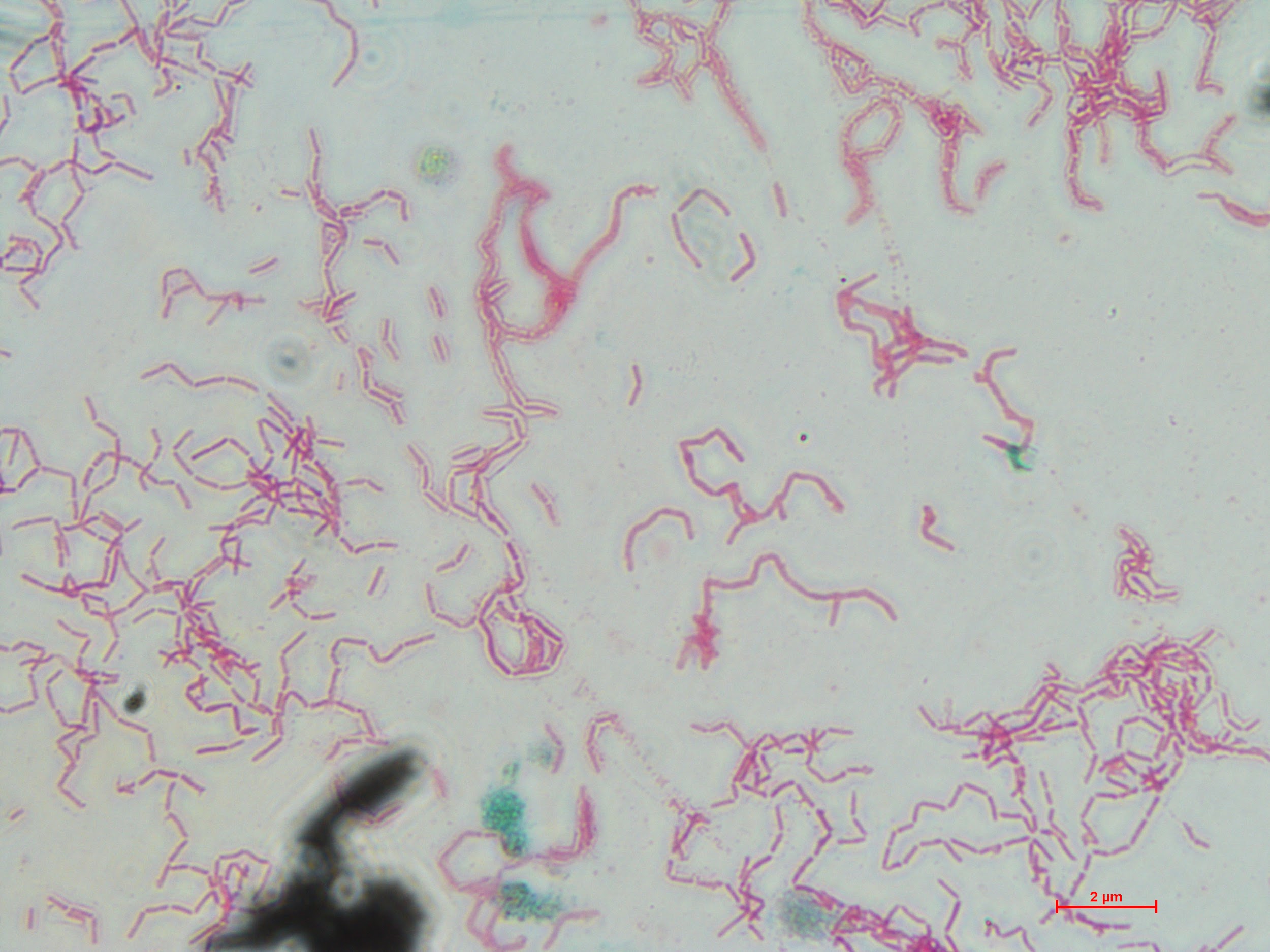 | 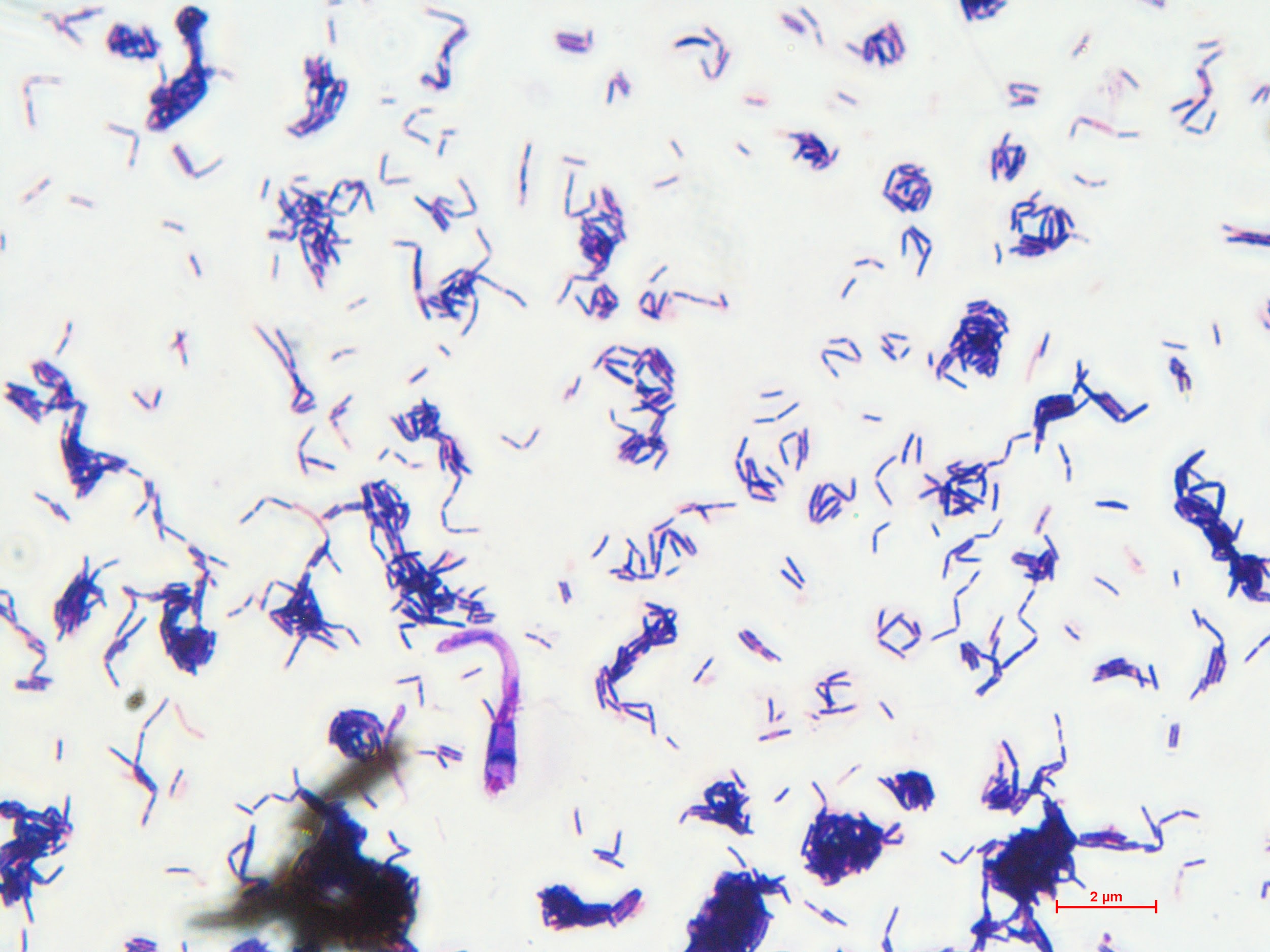 | P | **N** | N | N | N | N | N | **N** | N | P | P | N | P | N | N | N | N | N | N | N | N | N | N | N |
